# Review of the Applications of Density Functional Theory Calculations in Lithium–Sulfur Batteries

**DOI:** 10.3390/molecules31162750

**Published:** 2026-08-07

**Authors:** Ang Yu, Yingjie Ji, Guangrun Hu, Qi Zhang, Zhaodi Wang, Yi Zhang

**Affiliations:** 1 School of Energy Sciences and Engineering, Nanjing Tech University, Nanjing 211816, China; 202461208047@njtech.edu.cn (A.Y.); 202461208065@njtech.edu.cn (Y.J.); 202461208041@njtech.edu.cn (G.H.); 2 College of Electronic and Information Engineering, Anshun University, Anshun 561000, China; qzhang@mail.nwpu.edu.cn; 3 Key Laboratory for Light-Weight Materials, Nanjing Tech University, Nanjing 211816, China

**Keywords:** lithium-sulfur batteries, polysulfides, density functional theory, electrode

## Abstract

Lithium–sulfur (Li-S) batteries, featuring a superior theoretical energy density of 2600 Wh/kg and a high specific capacity of 1675 mAh/g for sulfur cathodes, have emerged as a promising candidate for next-generation, high-energy-density energy storage technologies. Nevertheless, their commercialization has been hindered by some bottlenecks, including the polysulfide (LiPSs) shuttle effect, severe volume expansion, and sluggish reaction kinetics. Density Functional Theory (DFT), serving as an atomic-scale computational tool, offers essential theoretical assistance for clarifying the mechanisms and guiding the precision design of S cathode of Li-S batteries. This review focuses on the role of DFT in the mechanism of polysulfide conversion and the shuttle effect. By simulating the adsorption energy, charge density distribution, and reaction pathways of LiPSs using DFT calculations, the key reaction steps of polysulfide conversion can be clearly identified. In addition, DFT calculations also play a vital role in the inhibition of lithium dendrites and the regulation of the solid-electrolyte interphase (SEI) film. This review suggests that DFT calculations could provide more applications in the development of Li-S batteries.

## 1. Introduction

Growing concerns over the energy crisis and climate change have spurred the demand for cost-effective energy storage systems with ultra-high energy density and ultralong cycling life [1,2,3,4,5,6]. The intrinsic limitations of lithium–ion batteries (LIBs) have impeded the fulfillment of the ever-growing demand for electric vehicles (EVs), portable electronics, and grid-scale energy storage systems [7,8,9]. Lithium–sulfur (Li-S) batteries exhibit a theoretical specific capacity of 1675 mAh g^−1^ and an energy density of 2600 Wh kg^−1^, which are substantially higher than those of lithium–ion batteries. Nevertheless, several critical challenges have hindered their practical implementation, including the dissolution and shuttle effect of lithium polysulfides, sluggish reaction kinetics, large volumetric expansion of sulfur, and lithium dendrite growth [10,11,12].

To address these challenges, conductive carbon materials, modified electrolytes, solid-state electrolytes (SSEs), and artificial solid electrolyte interphases (SEIs) have been employed individually or in combination to suppress lithium polysulfide dissolution, sulfur lithiation expansion, and lithium dendrite growth [13,14,15]. Despite significant experimental advances, mechanistic insights into the underlying causes of these persistent issues derived from simulations should also be harnessed to optimize battery design [16,17]. For instance, experiments have confirmed that the incorporation of sulfur with carbon nanomaterials can effectively mitigate lithium polysulfide dissolution, yet it remains challenging to elucidate how carbon nanomaterials modulate sulfur utilization through experimental approaches alone [18,19]. In addition, the uneven deposition of lithium ions on the anode surface induces lithium dendrite growth, which may pierce the separator and cause battery short-circuit and safety hazards.

DFT, a quantum mechanics-based electronic structure calculation method, is centered on describing the ground-state structures and properties of materials by solving the electron density of the system rather than the complex many-electron wavefunction [20,21]. Its theoretical foundation lies in the Hohenberg-Kohn theorems, which state that the ground-state properties of a system are uniquely determined by its ground-state electron density and that there exists a minimum value of the energy functional corresponding to the ground-state density [22]. It further simplifies the solution of many-electron problems to that of single-electron problems via the Kohn-Sham equations, where the approximate treatment of the exchange-correlation energy term ($E_{xc}\left[\rho \right]$)—such as local density approximation (LDA), generalized gradient approximation (GGA), and hybrid functionals—acts as the key factor governing computational accuracy [23,24]. DFT’s core advantage lies in balancing computational accuracy and efficiency: it can tackle complex systems containing hundreds to thousands of atoms and predict various material properties, including crystal structures, electronic band structures, reaction pathways, and adsorption energies [25,26,27]. In the materials science, the structures of composites play key roles in their performance [28,29,30,31,32,33]. Thus, DFT widely applied in the field of materials science. Yet this method suffers from inherent limitations: for instance, conventional DFT cannot accurately characterize strongly correlated systems, cannot directly address excited states, and its calculation results depend on the selection of functionals, which requires researchers to adapt according to the characteristics of the target system.

Recent DFT studies have provided scientific insights into the experimentally observed polysulfide immobilization, mitigation of volumetric expansion, and enhancement of battery capacity [34,35]. Furthermore, the dissolution of lithium polysulfides, shuttle effect, and lithium dendrite growth typically occur within overlapping time windows and thus cannot be investigated independently [36,37,38,39]. Multiphysics finite element analysis (FEA) enables the simultaneous characterization of electrodes and electrolytes during charge/discharge processes [40,41]. Moreover, electrochemical experiments involve time-consuming material preparation and the use of hazardous chemicals, whereas simulations require no additional materials or equipment and can generally be implemented in a short timeframe, thereby offering numerous advantages for reducing the development costs of lithium–sulfur (Li-S) batteries [42,43,44]. For instance, DFT simulations exhibit high efficiency in the screening of candidate materials and robust reliability in the prediction of their electrochemical performance, which drastically cuts down experimental costs [45,46,47,48].

This paper systematically reviews the latest research advances in electrochemical simulations for Li–S batteries and summarizes the applications of various simulation methods in cathodes, anodes, and electrolytes. All of the literature included in this review was retrieved from the Web of Science and Scopus databases within the time range of 2015–2026, using core keywords, including “lithium-sulfur battery”, “DFT”, “density functional theory”, “electrode interface”, “SEI film”, “polysulfide”, “adsorption binding energy”, and “density of states”. Duplicate and irrelevant studies were excluded after manual screening of titles, abstracts, and full texts. Different from previous reviews (e.g., [26]) that mainly focus on DFT applications in cathode materials while ignoring anode-related mechanisms, this work further supplements DFT simulation discussions on lithium dendrite growth and SEI evolution at lithium anodes and innovatively analyzes the applicable characteristics of various DFT functionals in Li–S systems, thereby providing a concise yet comprehensive reference for future electrochemical simulation research on Li–S batteries.

## 2. Principles of Density Functional Theory Simulations

DFT is a quantum mechanics-based electronic structure calculation method that focuses on describing and predicting the structures and properties of atoms, molecules, and condensed matter materials by solving the electron density of the system rather than the complex many-electron wavefunction. It has thus emerged as a core tool for investigating the microscopic mechanisms of substances in fields, including chemistry, physics, and materials science [49].

DFT simulations focus on three core aspects: the fundamental principles and energy components of the DFT method, as well as the types and applications of different exchange–correlation functionals. DFT enables the direct calculation of the microscopic properties of lithium–sulfur (Li-S) battery materials (e.g., sulfur cathodes, lithium anodes, lithium polysulfides, and electrolytes)—including electronic structures, chemical bonds, band gaps, and binding energies—thereby elucidating the intrinsic mechanisms governing material performance, such as the structural stability of lithium polysulfides and the electronic interactions at electrode–electrolyte interfaces [50].

The composition of the DFT energy functional is embodied in its total energy, which comprises four distinct components [51]

(1) $$E\left[\rho \right]=T_{KS}\left[\rho \right]+E_{ext}\left[\rho \right]+E_{H}\left[\rho \right]+E_{xc}\left[\rho \right]$$

where ρ denotes the electron density, the core variable in DFT; $T_{KS}\left[\rho \right]$ is the Kohn-Sham single-electron kinetic energy functional; $E_{ext}\left[\rho \right]$ is the electron-external field interaction energy functional (corresponding to the external potential); $E_{H}\left[\rho \right]$ is the classical Coulomb repulsion energy (Hartree energy) functional between electrons; and $E_{xc}\left[\rho \right]$ is the exchange-correlation energy functional, the core approximate term in DFT that describes the quantum exchange and correlation interactions among electrons.

The computational accuracy of DFT is governed by $E_{xc}\left[\rho \right]$, which, however, cannot be calculated exactly. Thus, various approximate models of exchange-correlation functionals have been developed to address this challenge.

The exchange-correlation energy functional for the local density approximation (LDA) is given by [52]:

(2) $$E_{xc}^{LDA}\left[\rho \right]=\int \varepsilon_{xc}^{LDA}\left(\rho \left(r\right)\right)d^{3}r$$

where $\varepsilon_{xc}^{LDA}\left(\rho \right)$ denotes the exchange-correlation energy density, which depends only on the electron density ρ at a given spatial point and represents the exchange-correlation energy per unit volume. The triple integral over the entire real space yields the total exchange-correlation energy of the system, corresponding to the volume integration of the exchange-correlation energy density at all spatial points.

LDA is founded on the key approximation that the electron density is uniformly distributed in space, such that the exchange-correlation energy at any spatial point can be calculated solely from the local electron density at that point. This approximation is therefore well-suited for systems with homogeneous electron density, such as metals. LDA exhibits the advantages of straightforward calculation and excellent numerical stability, yet it suffers from notable limitations for systems with inhomogeneous electron density (e.g., molecules and semiconductors): it underestimates chemical bond lengths and overestimates binding energies, severely underestimates the band gaps of semiconductors, and fails to capture long-range van der Waals forces, which results in substantial errors in the description of layered materials and molecular adsorption [53].

The exchange-correlation energy functional for the generalized gradient approximation (GGA) is given by [52]:

(3) $$E_{xc}^{GGA}\left[\rho \right]=\int \varepsilon_{xc}^{GGA}\left(\rho \left(r\right),\left|\nabla \rho \left(r\right)\right|\right)d^{3}r$$

Compared with LDA, $\varepsilon_{xc}^{GGA}$ depends not only on the local electron density ρ at a given spatial point but also on the gradient of the electron density $\left|\nabla \rho \right|$, namely the spatial variation rate of electron density in space. The general form of the GGA exchange energy is obtained by multiplying the LDA exchange energy by a gradient correction factor, while the GGA correlation energy is typically derived from the correlation energy of the uniform electron gas with the introduction of a correction term for the electron density gradient. The integration logic of GGA is consistent with that of LDA, yet it additionally accounts for the inhomogeneity of the electron density [54].

GGA is currently the most widely adopted functional in DFT simulations and can describe systems with inhomogeneous electron density. It exhibits excellent universality and moderate computational cost and yields more accurate predictions of bond lengths and binding energies for molecules and solids in comparison with LDA. Nevertheless, GGA still has inherent limitations: it underestimates the band gaps of semiconductors, cannot accurately characterize long-range van der Waals forces, and suffers from inadequate accuracy for strongly correlated systems.

The van der Waals-corrected functional (vdW-DF) is given by [51]:

(4) $$E_{xc}^{vdW\text{-}DF}\left[\rho \right]=E_{xc}^{GGA}\left[\rho \right]+E_{c}^{nl}\left[\rho \right]$$

Here, $E_{xc}^{GGA}[\rho ]$ denotes the GGA exchange-correlation energy, which describes short-range electronic interactions, and $E_{c}^{nl}\left[\rho \right]$ is the non-local correlation energy that accounts for long-range dispersion forces. The nonlocal correlation term is expressed as [51]:

(5) $$E_{c}^{nl}\left[\rho \right]=\frac{1}{2}\int \int n(r)\Phi \left(r,r^{\prime }\right)n(r^{\prime })drdr^{\prime }$$

where n(r) is the electron density and $\Phi \left(r,r^{\prime }\right)$ is a kernel function that depends on the density and its gradient. This kernel is derived from first principles rather than fitted to empirical data, distinguishing vdW-DF from empirical dispersion correction methods such as DFT-D3. It should be noted that the two strategies—empirical dispersion corrections and nonlocal vdW-DF functionals—differ in their theoretical foundations. Empirical corrections such as DFT-D3 add a pairwise interatomic correction to the GGA energy, whereas vdW-DF functionals incorporate dispersion effects directly into the exchange-correlation functional. The latter provides a more rigorous first-principles treatment of long-range correlation, while empirical corrections offer a computationally efficient alternative for a large-scale system.

vdW-DF is a DFT exchange-correlation energy model specifically designed to improve the description of long-range van der Waals forces—noncovalent weak interactions originating from the instantaneous polarization of electron clouds. Conventional LDA and GGA functionals adopt local or semi-local forms of exchange-correlation energy that depend only on the electron density or its gradient at a single spatial point and thus fail to capture such long-range, nonlocal interactions. This deficiency results in substantial computational errors in the description of layered materials (e.g., graphene stacking) and molecular adsorption systems (e.g., CO_2_ adsorption on solid surfaces). vdW-DF is therefore well suited for systems dominated by weak intermolecular interactions, such as the calculation of inter-layer stacking energies for graphene, MoS_2_, black phosphorus, and other layered nanomaterials [55].

The exchange-correlation energy functional for the meta-generalized gradient approximation (meta-GGA) is given by [52]:

(6) $$E_{xc}^{meta\text{-}GGA}\left[\rho \right]=\int \varepsilon_{xc}^{meta\text{-}GGA}\left(\rho \left(r\right),\left|\nabla \rho \left(r\right)\right|,\tau \left(r\right)\right)d^{3}r$$

where $\rho \left(r\right)$ is the electron density (the fundamental variable); $\left|\nabla \rho \left(r\right)\right|$ denotes the electron density gradient (a correction term inherited from GGA); $\tau \left(r\right)$ is the Kohn-Sham (KS) single-electron kinetic energy density (the core new term introduced in meta-GGA); and $\varepsilon_{xc}^{meta\text{-}GGA}$ is the meta-GGA exchange-correlation energy density, which is a function of the three aforementioned variables.

The limitation of GGA lies in its sole reliance on electron density and its gradient to describe electronic distribution, which fails to reflect the electronic dynamic states (e.g., the occupation of local orbitals and the spatial distribution of electron kinetic energy). As an advanced exchange-correlation energy functional in DFT with higher accuracy than GGA, meta-GGA’s core improvement is the further introduction of the kinetic energy density τ(r) on the basis of GGA’s two key variables (ρ(r) and |∇ρ(r)|), thus enabling a more refined description of the local electronic behavior and electron density inhomogeneity [56].

meta-GGA captures the real electronic behavior in the description of exchange-correlation energy more accurately and has thus emerged as the primary functional for high-accuracy calculations in materials science and chemistry. For research scenarios requiring precise characterization of adsorption energies, reaction pathways, and electronic structures—such as lithium–sulfur (Li-S) battery systems—meta-GGA functionals (e.g., SCAN) represent a superior alternative to GGA. They can effectively balance computational accuracy and efficiency without the need for additional complex correction terms [57].

Despite their improved accuracy, meta-GGA functionals are not universally superior to GGA. For certain systems, meta-GGA may overcorrect lattice constants or produce less accurate total energies compared with GGA, and their computational cost is noticeably higher, particularly for large unit cells. Moreover, the performance of meta-GGA functionals can vary significantly depending on the specific material and property under investigation. Therefore, the choice of meta-GGA should be justified by systematic benchmarking against experimental data or higher-level theoretical methods, rather than assumed a priori to be optimal for all systems.

In addition to these semi-local and meta-GGA functionals, hybrid functionals provide another route for improving the accuracy of electronic structure calculations. The exchange-correlation energy functional for hybrid functionals is given by:

(7) $$E_{xc}^{hybrid}\left[\rho \right]=aE_{x}^{HF}\left[\rho \right]+\left(1-a\right)E_{x}^{GGA}\left[\rho \right]+E_{c}^{GGA}\left[\rho \right]$$

where $E_{x}^{HF}$ is the exact Hartree–Fock exchange energy, $E_{x}^{GGA}$ and $E_{c}^{GGA}$ are the GGA exchange and correlation energies, respectively, and a is the fraction of Hartree–Fock exchange mixed into the functional. For example, the PBE0 functional adopts a fixed mixing ratio of a = 0.25, while the HSE06 functional employs a range-separated approach that includes HF exchange only for short-range electron interactions. Hybrid functionals significantly improve the accuracy of band gap predictions for semiconductors and insulators compared with GGA. However, their high computational cost limits their application to small systems or benchmark calculations, while GGA-based methods remain the practical choice for large-scale interfaces and adsorption studies in Li–S battery research.

For systems where strong electron correlation plays a critical role, additional corrections beyond hybrid functionals are required. Functionals for Strongly Correlated Systems:

The most commonly used strongly correlated corrected functionals include LDA+U and GGA+U, with GGA+U taken as a typical example [52]:

(8) $$E_{xc}^{GGA+U}\left[\rho \right]=E_{xc}^{GGA}\left[\rho \right]+E_{Hubbard-U}\left[\rho \right]$$

where $E_{xc}^{GGA}\left[\rho \right]$ denotes the conventional GGA exchange-correlation energy (e.g., PBE), and $E_{Hubbard-U}\left[\rho \right]:$ refers to the Hubbard U correction term.

The core of strongly correlated functionals lies in the additional introduction of a local Coulomb repulsion correction term into the exchange-correlation energy of conventional DFT, which enables the explicit description of strong electronic correlation effects in localized orbitals (e.g., d-orbitals of transition metals and f-orbitals of rare earth elements). Such functionals are well-suited for the electronic structure calculations of transition metal oxides (MnO, FeO, CoO, NiO), rare earth oxides (CeO_2_, La_2_CuO_4_), and other related materials [58].

The Hubbard U parameter is not a universally transferable value; its selection significantly affects the calculated electronic structure, band gaps, and total energy. Common approaches for determining U include empirical fitting to experimental data, linear response theory within DFT, and self-consistent methods such as ACBN0. The choice of U requires careful benchmarking against experimental or higher-level theoretical results, as overestimated or underestimated U values may lead to qualitatively incorrect predictions of electronic properties. In addition, for transition metal oxides and other magnetic systems, spin-polarized calculations are indispensable, as neglecting spin polarization can yield fundamentally erroneous electronic structures. The application of DFT+U should therefore be accompanied by systematic convergence tests with respect to the U value and a clear justification of the chosen parameter.

The exchange-correlation functionals of DFT are focused on the core objective of accurately describing electronic exchange and correlation effects, forming a hierarchical framework based on the complexity of input variables and the degree of approximation: starting from the LDA, which relies solely on electron density and is applicable to simple metals with homogeneous electron density, featuring an ultra-low computational cost yet limited accuracy; to the GGA, which incorporates the electron density gradient and is represented by the PBE functional, boasting excellent universality and moderate computational cost as the default choice for conventional systems; then to meta-GGA, with the additional introduction of kinetic energy density, achieving a marked improvement in accuracy to capture more refined local electronic behavior [59]. In addition, special functionals optimized for specific effects (e.g., vdW-DF) augment the description of long-range van der Waals forces. Furthermore, LDA+U and GGA+U modify strongly correlated systems via the Hubbard U term to accurately capture dynamic strong correlation effects, thus providing a flexible and highly efficient theoretical tool for atomic-scale material design and property prediction [60].

To ensure the reliability of DFT predictions, rigorous method validation is essential. For plane-wave DFT calculations, convergence tests with respect to cutoff energy and k-point mesh must be performed, and the thresholds for atomic force and energy convergence should be carefully specified. Additionally, calculated properties—such as lattice constants, band gaps, and adsorption energies—should be compared with available experimental data or higher-level theoretical benchmarks (e.g., GW or hybrid functional calculations) to assess the accuracy of the chosen exchange-correlation functional. Systematic benchmarking across multiple functionals is recommended for properties that are sensitive to the choice of functional. Transparent reporting of these validation procedures enhances the credibility and reproducibility of DFT-based studies.

## 3. Applications of Density Functional Theory in Lithium–Sulfur (Li-S) Batteries

Sulfur features low cost, natural abundance, and environmental benignity [61]. However, its application in lithium–sulfur batteries is hindered by several critical challenges, including lithium polysulfide dissolution, low electrical conductivity, and volumetric expansion induced by lithiation [62]. A variety of computational methods have been employed to simulate the cathodes of Li-S batteries [63,64]. DFT enables the fundamental understanding of lithium polysulfide adsorption, cathodic electrochemistry, and battery degradation mechanisms in such systems.

### 3.1. Applications for Adsorption Binding Energy

The conversion between sulfur and Li_2_S generates a series of long-chain lithium polysulfides (Li_2_S*_n_*, *n* = 4–8), which readily dissolve into the electrolyte and migrate toward the anode, thereby resulting in rapid capacity fading and severe self-discharge [65]. To suppress the dissolution and migration of lithium polysulfides, numerous strategies have been developed, including functional sulfur hosts, functional interlayers, functional binders, modified electrolytes, and modified separators [66,67]. In this section, the calculation of adsorption binding energies for sulfur hosts is discussed. The essence of adsorption binding energy lies in the energy variation during the adsorption process, which is used to quantify the strength of intermolecular interactions between adsorbates and adsorbents [68]. In DFT calculations, the binding energy (Eb) is typically defined as the internal energy change (ΔU) at 0 K in vacuum. It should be noted that a negative Eb does not strictly equate to spontaneity under realistic electrochemical conditions, as the true thermodynamic criterion is the Gibbs free energy change (ΔG), which must account for entropic losses (TΔS), solvation effects, and the applied electrode potential. Nevertheless, the calculated Eb values serve as a useful trend indicator for comparing the relative adsorption affinities of different host materials. Within a reasonable range, a more negative Eb generally indicates stronger immobilization of LiPSs [69,70,71].

However, according to the Sabatier principle, excessively strong adsorption (e.g., Eb < −3.0 eV) can be detrimental, as it may impede the desorption and subsequent conversion of intermediates, leading to active site poisoning and sluggish redox kinetics. Therefore, an optimal adsorption strength that balances trapping capability and catalytic convertibility is desirable.

DFT serves as the core theoretical tool for the calculation of adsorption binding energies and the analysis of interaction mechanisms in Li-S batteries. By accurately quantifying binding energy values of different systems and selecting appropriate functionals, it addresses key issues in Li-S batteries in a targeted manner, including the shuttle effect, sluggish reaction kinetics, and poor interfacial stability [72]. Combined with the adaptation of functionals such as vdW-DF and GGA+U to different types of adsorption interactions, DFT guides modification strategies, including heteroatom doping and defect engineering, thereby achieving effective anchoring of lithium polysulfides (LiPSs) to suppress the shuttle effect. It can also screen catalysts capable of reducing dissociation energy barriers by analyzing the binding energies and energy barriers of Li_2_S nucleation and dissociation [73,74,75], thus accelerating the progress of sulfur redox reactions [76,77]. Gong et al. [78] prepared graphene quantum dots/sulfur (GQDs/S) composites as cathode materials for Li-S batteries. DFT simulations demonstrated that the multiple polar functional groups in GQDs can provide more active adsorption sites for LiPSs; thus, these materials significantly enhance the chemical adsorption of LiPSs and remarkably suppress the shuttle effect [79].

**Figure 1:**
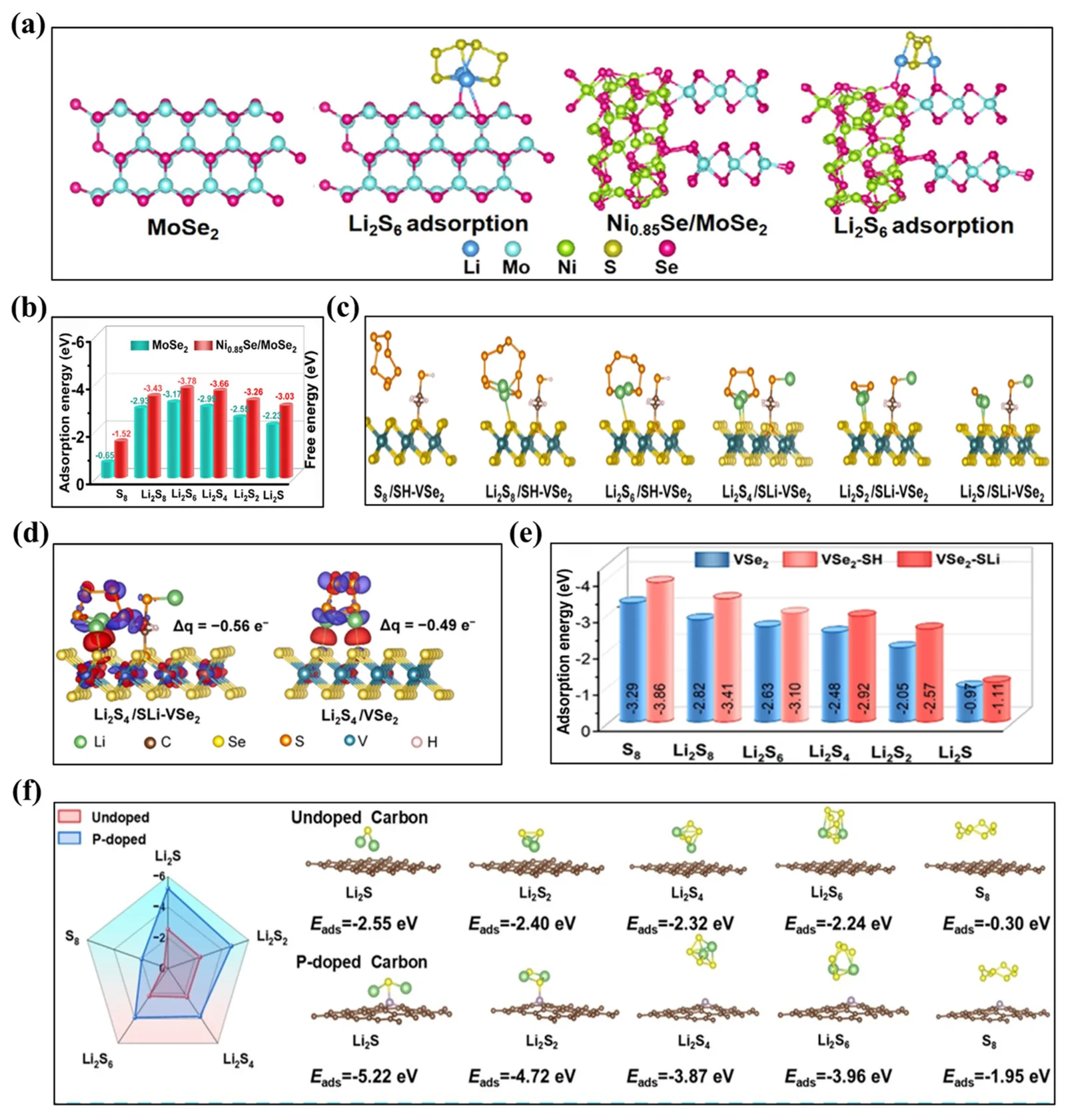
(**a**) Optimized adsorption geometries [81]. (**b**) adsorption energies of LiPSs [81]. Copyright 2025, Wiley. (**c**) Optimized geometric configuration of S species adsorbed on SLi-VSe_2_/SLi-VSe_2_ [82]. (**d**) Charge-density difference and interfacial electron transfer of Li_2_S_4_ interacting with SH-VSe_2_ (**left**) and VSe_2_ (**right**). Red and blue isosurfaces represent electron accumulation and electron depletion at an isosurface cutoff of 0.002 e^−^/Bohr3 [82]. (**e**) Adsorption energies of various sulfur species [82]. Copyright 2025, Elsevier. (**f**) Radar map of binding energies and optimized geometries of the Li_2_Sn (*n* = 1, 2, 4, and 6), as well as the S_8_ molecule adsorbed on the P-doped (or undoped) carbon, with corresponding values of adsorption energy listed below the models. (Lithium, sulfur, phosphorus, and carbon atoms are marked with green, yellow, violet, and brown, respectively) [83]. Copyright 2025, Elsevier.

Figure 1a presents the atomic structures of pure MoSe_2_ and Ni-doped Ni_0.85_Se/MoSe_2_. DFT calculations were employed to simulate the atomic-scale material structures and predict the most stable adsorption sites and configurations of Li_2_S_6_ on the material surfaces, thus providing fundamental structural models for subsequent energy calculations. The bar chart in Figure 1b compares the adsorption energies of sulfur species (S_8_, Li_2_S_6_, Li_2_S, etc.) on pure MoSe_2_ and Ni_0.85_Se/MoSe_2_. The data show that the adsorption energies of the Ni-doped material for all sulfur species are significantly enhanced, which represents the core approach for DFT to evaluate a material’s capability to suppress the shuttle effect. Figure 1c depicts the catalytic conversion pathways, revealing the chemical nature of the adsorption interaction (strong electronic interactions). The charge density difference analysis (Figure 1d) demonstrates enhanced charge accumulation (red) and depletion (blue) at the Li_2_S_4_/SLi-VSe_2_ interface in comparison with Li_2_S_4_/VSe_2_, indicating a stronger interfacial interaction. Bader charge quantification confirms that the electron transfer from Li_2_S_4_/SLi-VSe_2_ (0.56 e per supercell) is higher than that to VSe_2_ (0.49 e per supercell). For the adsorption energies across the lithiation process, Figure 1e verifies that SLi-VSe_2_/SH-VSe_2_ exhibits consistently stronger binding interactions, which is consistent with experimental adsorption tests and confirms its excellent shuttle effect suppression performance. DFT calculations fulfill critical roles in atomic structure modeling, adsorption energy quantification, reaction thermodynamics analysis, and electron transfer characterization, delivering precise theoretical underpinnings for material design and reaction mechanism investigations in Li-S batteries across multiple scales, spanning from the elucidation of microscopic mechanisms to the assessment of material performance. Muhammad et al. [80] probed the polysulfide anchoring capabilities of monolayer porous triphenylene-graphdiyne (TPGDY), boron-doped graphdiyne (BGDY), and nitrogen-doped graphdiyne variants (NGDY-C_18_N_6_, NGDY-C_24_N_4_, NGDY-C_36_N_6_) via DFT-based computations. Their findings demonstrate that the BGDY, TPGDY, NGDY-C_18_N_6_, and NGDY-C_36_N_6_ substrates exhibit robust adsorption energies toward lithium polysulfide (Li_2_S_n_) molecules, thereby inhibiting Li_2_S_n_ leaching into the electrolyte and aiding in the suppression of the polysulfide shuttle effect.

**Figure 2:**
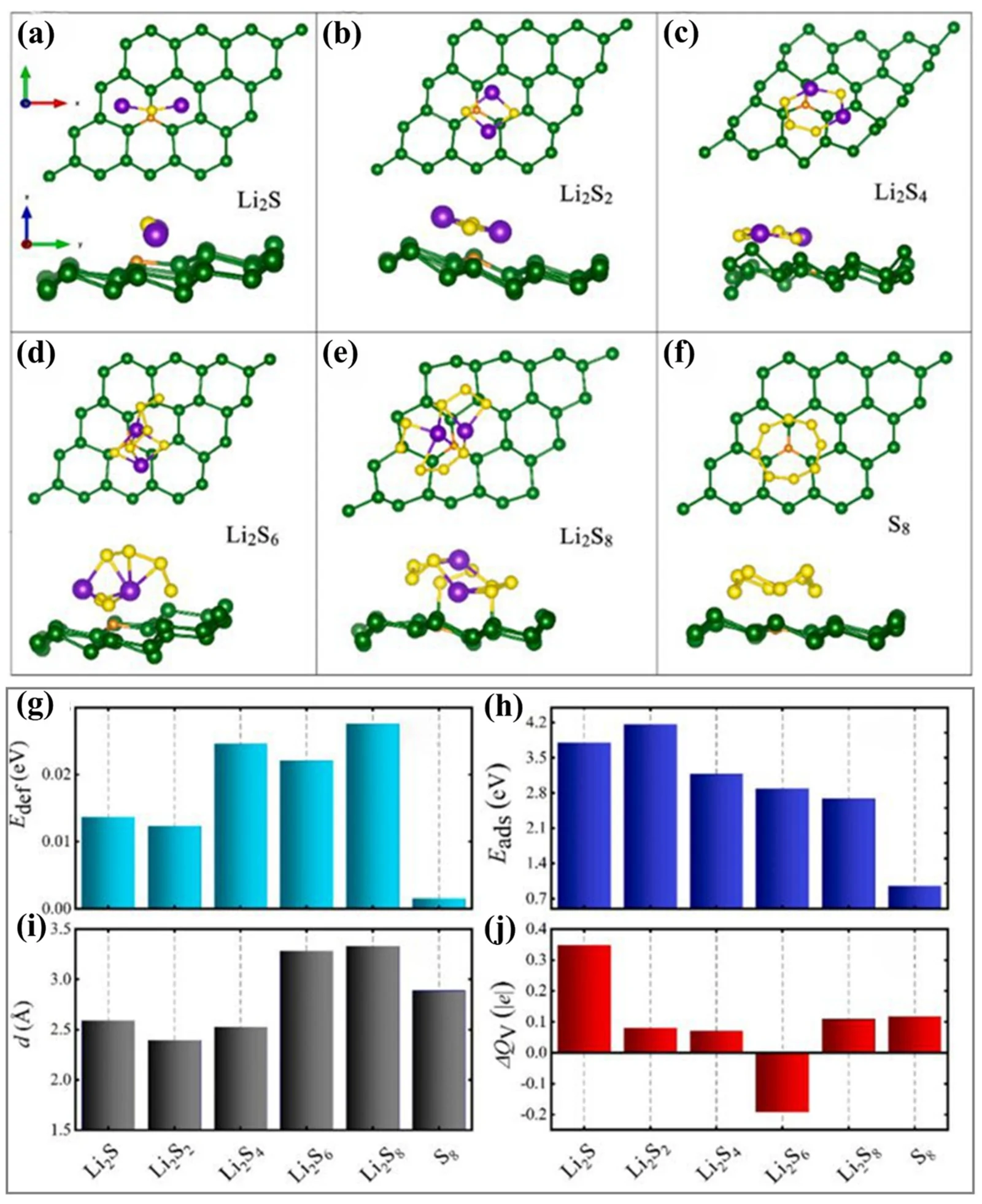
(**a**–**f**) Side and top views of the most stable configurations of LiPSs adsorbed on B-2DGe and (**g**–**j**) deformation energy (Edef), adsorption energy (Eads), average bond distance (d), and charge transfer (ΔQ_V_) [27]. Copyright 2024, Elsevier.

Figure 2 systematically illustrates the adsorption behaviors of lithium polysulfides (Li_2_S_n_, *n* = 1, 2, 4, 6, 8) and elemental sulfur (S_8_) on the surface of B-doped two-dimensional graphdiyne (B-2DGe) via DFT calculations by Rosas et al. [27]. Figure 2a–f present the DFT-optimized stable adsorption configurations of various sulfur species from side and top views, visually revealing the binding characteristics and adsorption mode differences between sulfur species with different chain lengths and B-doped sites. This clearly reflects the material’s preference for anchoring sites and spatial interaction modes toward the full range of sulfur species, spanning from short-chain Li_2_S to long-chain Li_2_S_8_ and elemental sulfur S_8_.

Figure 2g quantifies the structural deformation degree of sulfur species after adsorption by means of deformation energy, which reflects their structural stability and activation difficulty. The adsorption energies in Figure 2h directly characterize the interaction strength between the material and various sulfur species, confirming the material’s robust anchoring capability for polysulfides with different chain lengths. Figure 2i displays the average bond lengths, illustrating the changes in S–S bonds after adsorption to reflect the activation degree of polysulfides. Figure 2j shows the charge-transfer amounts, revealing the direction and intensity of electron flow during the adsorption process. This indicates that the interaction between LiPSs and B-2DGe facilitates the structural deformation of polysulfides. In addition, the final configurations of polysulfides are significantly different from their original forms, which further demonstrates the strong interfacial interaction between polysulfides and B-2DGe.

**Figure 3:**
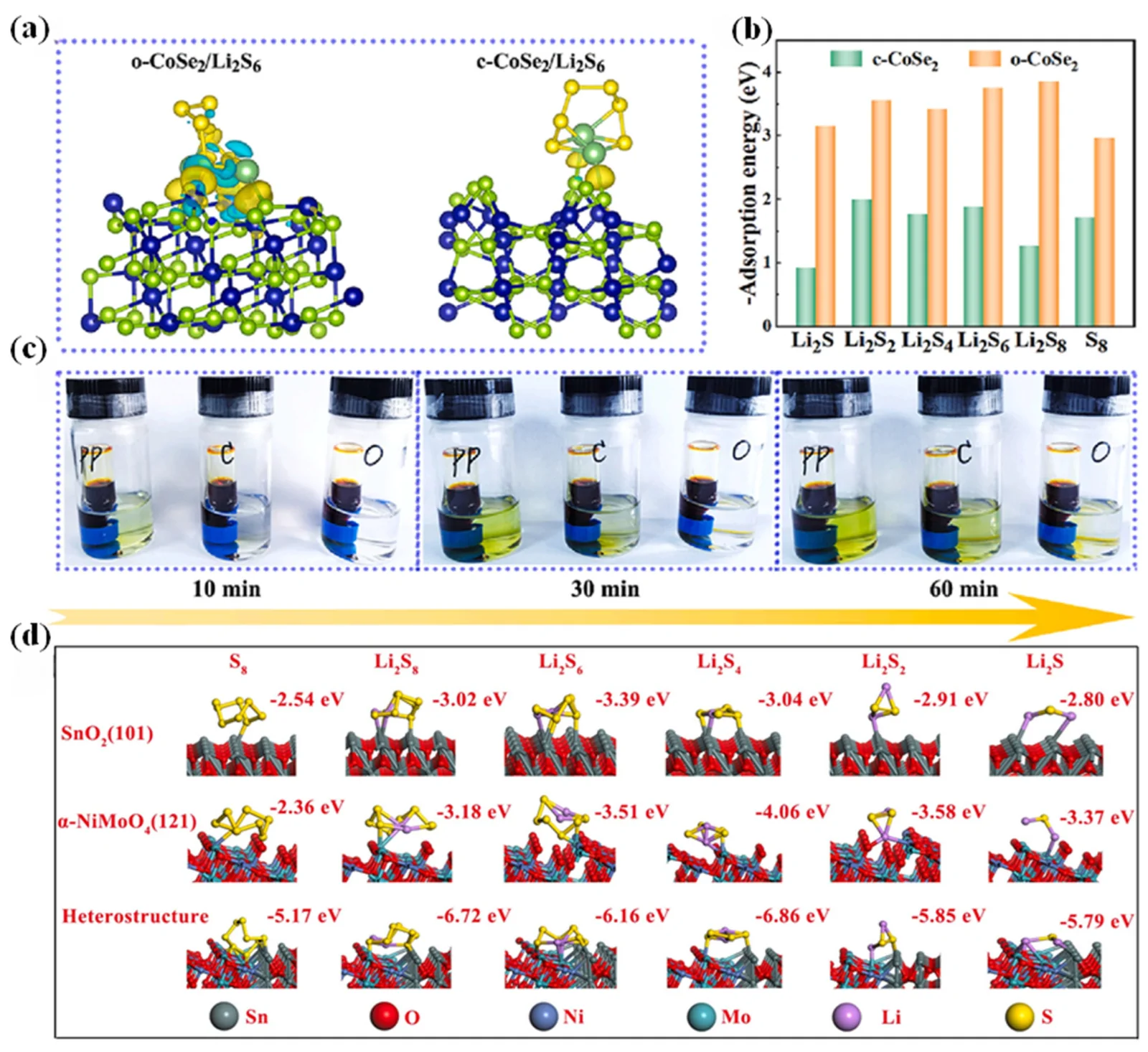
Polysulfide adsorption on catalyst surfaces. (**a**) Charge density difference of o-CoSe_2_/Li_2_S_6_ and c-CoSe_2_/Li2S_6_ adsorption systems. The isosurface level is 0.003 electrons/bohr3; blue and yellow isosurfaces denote negative and positive regions, respectively. (**b**) Adsorption energies between different catalysts and sulfur species. (**c**) Photographs of polysulfide diffusion from concentrated Li_2_S_6_ to pure DOL/DME solvent through clean PP separator (marked as PP), c-CoSe_2_@PP (marked as C), and o-CoSe_2_@PP (marked as O), respectively [84]. Copyright 2023, Elsevier. (**d**) Optimized adsorption configurations and binding energies of LiPSs on α-NiMoO_4_, SnO_2_, and SnO_2_@α-NiMoO_4_ heterostructures [85]. Copyright 2026, Elsevier.

Figure 3 illustrates the assessment of interactions between lithium polysulfides (LiPSs) and CoSe_2_ samples via DFT calculations, as reported by Liu et al. [84]. The orthorhombic CoSe_2_ (o-CoSe_2_) and cubic CoSe_2_ (c-CoSe_2_) catalysts were modeled using supercells with lattice parameters of 12.2 Å × 13.1 Å × 21.9 Å and 11.7 Å × 13.1 Å × 22.5 Å, respectively, coupled with a 15 Å vacuum slab. Figure 3a displays the charge density difference of the adsorption systems, where a distinct variation in charge density distribution is evident at the LiPSs-catalyst interfaces. In contrast to the LiPSs/o-CoSe_2_ adsorption system, charge redistribution at the LiPSs/c-CoSe_2_ interface is less pronounced, indicating a stronger interfacial interaction between LiPSs and o-CoSe_2_. Figure 3b presents the quantitative assessment of adsorption energies between sulfur species and the two CoSe_2_ catalysts.

Negative adsorption energy values denote a spontaneous adsorption process, with a larger absolute value reflecting a stronger interaction. The calculated adsorption energies of Li_2_S, Li_2_S_2_, Li_2_S_4_, Li_2_S_6_, Li_2_S_8_, and S_8_ on the c-CoSe_2_ surface are −0.922, −1.997, −1.763, −1.877, −1.270, and −1.714 eV, respectively, whereas those on the o-CoSe_2_ surface are −3.143, −3.558, −3.418, −3.750, −3.848, and −2.958 eV, respectively. These results verify that LiPSs exhibit substantially stronger adsorption affinity toward o-CoSe_2_ than toward c-CoSe_2_. The visualized adsorption tests in Figure 3c further confirm the excellent interception and capture capability of the o-CoSe_2_ catalyst for LiPSs. Figure 3d outlines systematic DFT computations conducted by Ye et al. [85] on α-NiMoO_4_, SnO_2_, and their SnO_2_@α-NiMoO_4_ heterostructure systems, intended to clarify the synergistic adsorption and catalytic mechanism of the SnO_2_@α-NiMoO_4_ heterostructure, as well as its regulatory role in reaction kinetics. This figure depicts the optimized interaction configurations and binding energy distributions (from S_8_ to Li_2_S) between the three material systems and sulfur species. Quantitative analysis reveals that the SnO_2_@α-NiMoO_4_ heterostructure exhibits markedly higher adsorption energies (−5.17 to −6.86 eV) for various LiPSs intermediates compared to pure SnO_2_ (−2.54 to −3.39 eV) and pristine α-NiMoO_4_ (−2.36 to −4.06 eV). This confirms that the synergistic interplay of the heterointerface significantly enhances the chemical anchoring of LiPSs, thereby effectively suppressing the polysulfide shuttle effect.

### 3.2. Applications of Band Structure and Decomposition Energy Barrier

The band structures and decomposition energy barriers calculated via DFT serve as core theoretical tools for driving the performance breakthrough of lithium–sulfur batteries from the dual dimensions of electronic level to reaction process, and their value is reflected not only in the analysis of micro-mechanisms but also in the direct guidance of the direction of experimental research and development [86,87].

The projected band structure analysis of DFT can also accurately reveal the orbital mechanism of electron transport: for instance, in the band structure of N-doped carbon, the 2p orbitals of N and C hybridize at the Fermi level [88], forming continuous electron transport channels. This not only ensures rapid electron transfer but also enhances the adsorption of lithium polysulfides, thus achieving the synergistic optimization of multiple functions [89].

**Figure 4:**
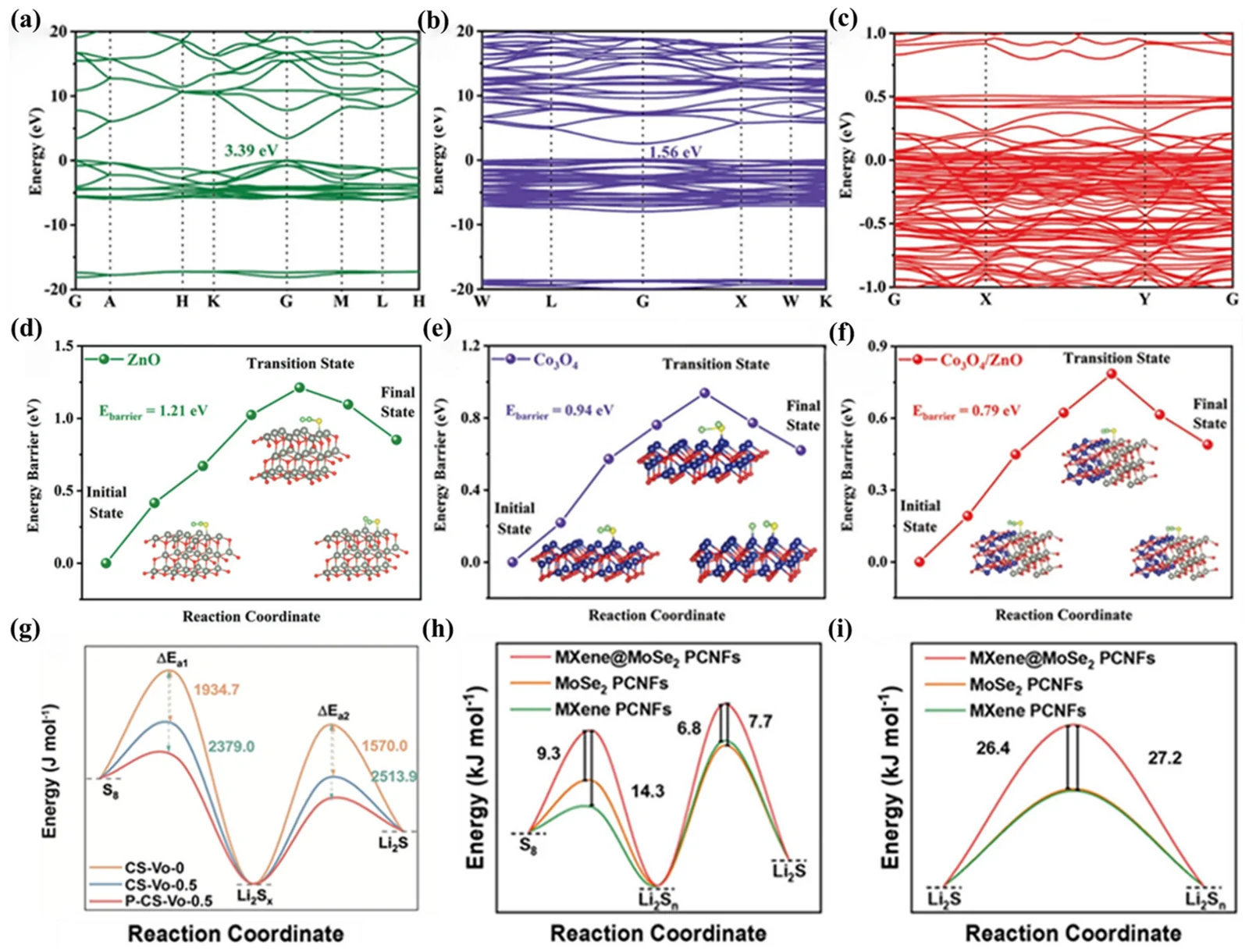
Band structures of (**a**) ZnO, (**b**) Co_3_O_4_, and (**c**) Co_3_O_4_/ZnO [95]. Energy profiles of the decomposition barriers of Li_2_S on (**d**) ZnO, (**e**) Co_3_O_4_, and (**f**) Co_3_O_4_/ZnO heterointerface (insets: the initial, transition, and final structures) [95]. Copyright 2023, Wiley. (**g**) Relative activation energies of the two sulfur cathodes [96]. Copyright 2025, Springer. (**h**,**i**) Activation energies of the precipitation and dissolution of Li_2_S [97]. Copyright 2025, Wiley.

Figure 4a–c show the band structures of different materials. DFT can analyze the electron transport properties of materials by calculating the energy level distribution and band gap of electrons. Generally, a smaller band gap suggests a lower transition resistance of electrons between the valence band and conduction band, which may indicate potentially better intrinsic conductivity [90,91,92]. However, it should be noted that the actual electrical conductivity of a material is governed by multiple factors, including carrier concentration, mobility, defect states, and temperature, rather than solely by the band gap. Therefore, the band gap obtained from DFT calculations serves as a qualitative indicator for comparing the relative electronic structures of different materials but does not directly quantify their absolute conductivity. Such calculations can help distinguish the electron transport capabilities of different materials and avoid the attenuation of rate performance caused by the selection of electrode materials with insufficient conductivity. The band gap of the material corresponding to Figure 4a is 3.39 eV and that of Figure 4b is 1.56 eV, suggesting that Co_3_O_4_ may have a narrower band gap and thus potentially better intrinsic conductivity compared with ZnO.

Figure 4d–f display the reaction energy barrier curves for the transformation of sulfur species in the ZnO, Co_3_O_4_, and Co_3_O_4_/ZnO composite systems. Based on DFT calculations, the geometric optimization of the atomic structures of the initial state, transition state, and final state was first completed, and then the reaction energy barriers of each system were quantified via transition state search. For example, the energy barrier on the ZnO surface is 1.21 eV, that on the Co_3_O_4_ surface is 0.94 eV, and that of the composite system is as low as 0.79 eV. The reduction of the energy barrier implies a decrease in the kinetic resistance of sulfur species transformation. This calculation clarifies the promotion effect of the composite catalyst on the reaction rate, provides accurate kinetic parameters for the design of high-efficiency catalytic systems, and helps break through the reaction rate bottleneck of lithium–sulfur batteries [93].

Figure 4g–i correspond to carbon materials with different defect concentrations, MoSe_2_-based composite systems, and MXene-based composite systems, respectively. DFT was used to calculate the free energy changes during the sulfur reduction (S_8_→Li_2_S) and sulfur oxidation (L_i2_S→Li_2_S_x_) processes. By comparing the free energy barriers and reaction enthalpy changes in different material systems, the thermodynamic promotion effect of materials on the entire sulfur redox process was evaluated. For example, the introduction of defects in modified carbon materials reduces the free energy barrier of the reaction [93], and the composite of MXene and MoSe_2_ makes the free energy change of Li_2_S decomposition more gentle. These results elucidate the regulatory mechanism of material modification on the thermodynamic feasibility of the reaction.

**Figure 5:**
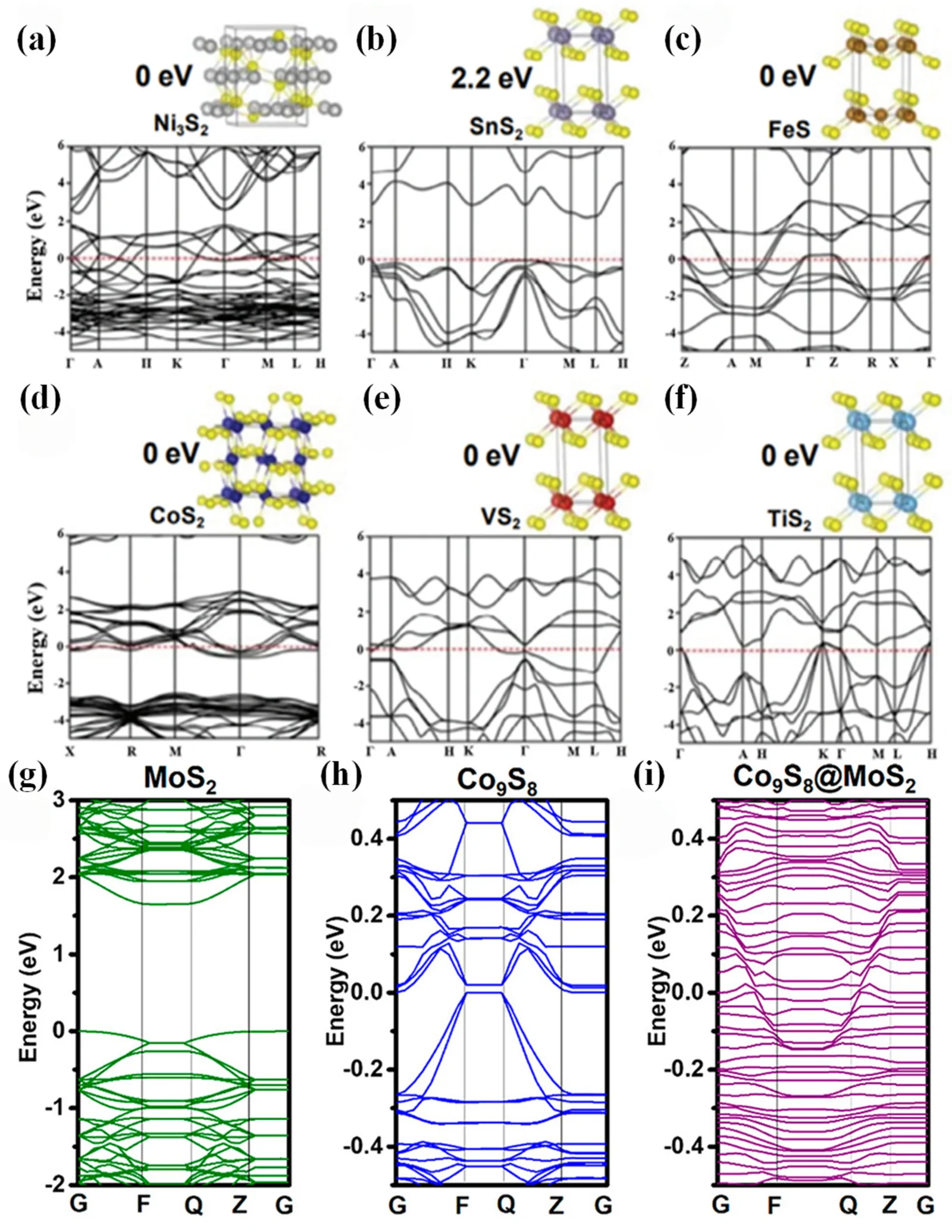
Crystal structures and calculated band structures for bulk phases of (**a**) Ni_3_S_2_, (**b**) SnS_2_, (**c**) FeS, (**d**) CoS_2_, (**e**) VS_2_, and (**f**) TiS_2_. The red dashed lines mark the Fermi energy level [94]. Copyright 2017, National Academy of Sciences. Calculated band structures of (**g**) MoS_2_, (**h**) Co_9_S_8_, and (**i**) Co_9_S_8_@MoS_2_ [98]. Copyright 2020, American Chemical Society.

Figure 5a–f show the electronic band structure calculations performed by Zhou et al. [94] using the Heyd, Scuseria, and Ernzerhof (HSE06) hybrid functional. The calculations indicate that the band gaps of VS_2_, CoS_2_, TiS_2_, FeS_2_, and NiS_2_ are 0 eV, suggesting that they possess metallic character, which is generally favorable for electrical conductivity. In fact, NiS_2_, FeS_2_, and CoS_2_ are metallic materials.

This functional is particularly successful not only in describing band gaps but also in characterizing the ground-state properties of the entire material. Figure 5g–i present the calculations of the band structures of MoS_2_, Co_9_S_8_, and Co_9_S_8_@MoS_2_ conducted by Li et al. [98]. As shown in Figure 5g, pristine MoS_2_ exhibits semiconductor characteristics with a direct band gap of 2.0 eV. The band structure of Co_9_S_8_ is displayed in Figure 5h, indicating that Co_9_S_8_ has metallic properties. It can be seen from Figure 5i that after the introduction of Co_9_S_8_, the band structure of Co_9_S_8_ is well retained; meanwhile, the energy level separation becomes more distinct, and there are more impurity energy levels in the band gap than those of Co_9_S_8_, which also illustrates that the metallic properties of Co_9_S_8_@MoS_2_ are improved. The HSE06 hybrid functional is applicable to a wider range of systems, but a trade-off is required in terms of high computational cost. To accurately determine the electronic properties, the band structures of sulfur hosts are usually calculated via DFT simulations, using either the DFT+U method or the HSE06 functional.

Overall, DFT calculations play a core role as a tool for simulating the atomic structures, analyzing the electronic band structures, and quantifying the reaction energy barriers and free energies of lithium–sulfur batteries. From the three dimensions of electron transport, kinetic rate, and thermodynamic trend, they provide precise atomic-scale theoretical support for the screening of electrode materials, design of catalysts, and clarification of reaction mechanisms in lithium–sulfur batteries [99,100].

### 3.3. Application in the Calculation of Charge Distribution and Density of States

In DFT investigations of lithium–sulfur (Li-S) batteries, the density of states (DOS) and charge distribution represent core analytical tools for deciphering electronic structures and microscopic interaction mechanisms [101]. DOS characterizes the energy-dependent distribution of electronic states, thereby revealing orbital hybridization, electrical conductivity, and the location of catalytically active sites. In contrast, charge distribution emphasizes spatial localization and dynamic transfer of electrons.

Together, DOS and charge distribution in DFT frameworks function as critical analytical pillars for unraveling the electronic structure and interfacial interaction mechanisms in Li-S batteries. These two approaches probe electronic behaviors from complementary energy and spatial perspectives, collectively supporting the rational optimization of electrode materials, high-throughput catalyst screening, and the enhancement of interfacial stability [102]. Leveraging total density of states (TDOS), projected density of states (PDOS), and local density of states (LDOS), DOS analysis facilitates the assessment of electrode conductivity and the distribution of electronic states near the Fermi level. It further reveals the orbital hybridization between electrode materials and lithium polysulfides (LiPSs)—as indicated by PDOS overlap—quantifies catalytic activity via the d-band center model, and traces the evolution of electronic states during Li_2_S nucleation and dissociation [103]. Via techniques such as charge density difference (Δρ) and Bader charge analysis, charge distribution analysis intuitively differentiates between physical and chemical adsorption events, quantifies the magnitude of charge transfer, informs charge modulation strategies for heteroatom doping and defect engineering, and evaluates the rationality of charge transfer at the electrode–electrolyte interface to safeguard interfacial stability [104]. The synergistic integration of DOS and charge distribution analyses enables a comprehensive elucidation of intrinsic microscopic interactions, including orbital hybridization and charge transfer dynamics. This integration delivers quantitative, electronic-level insights that underpin strategies to mitigate the polysulfide shuttle effect and enhance reaction kinetics [105].

**Figure 6:**
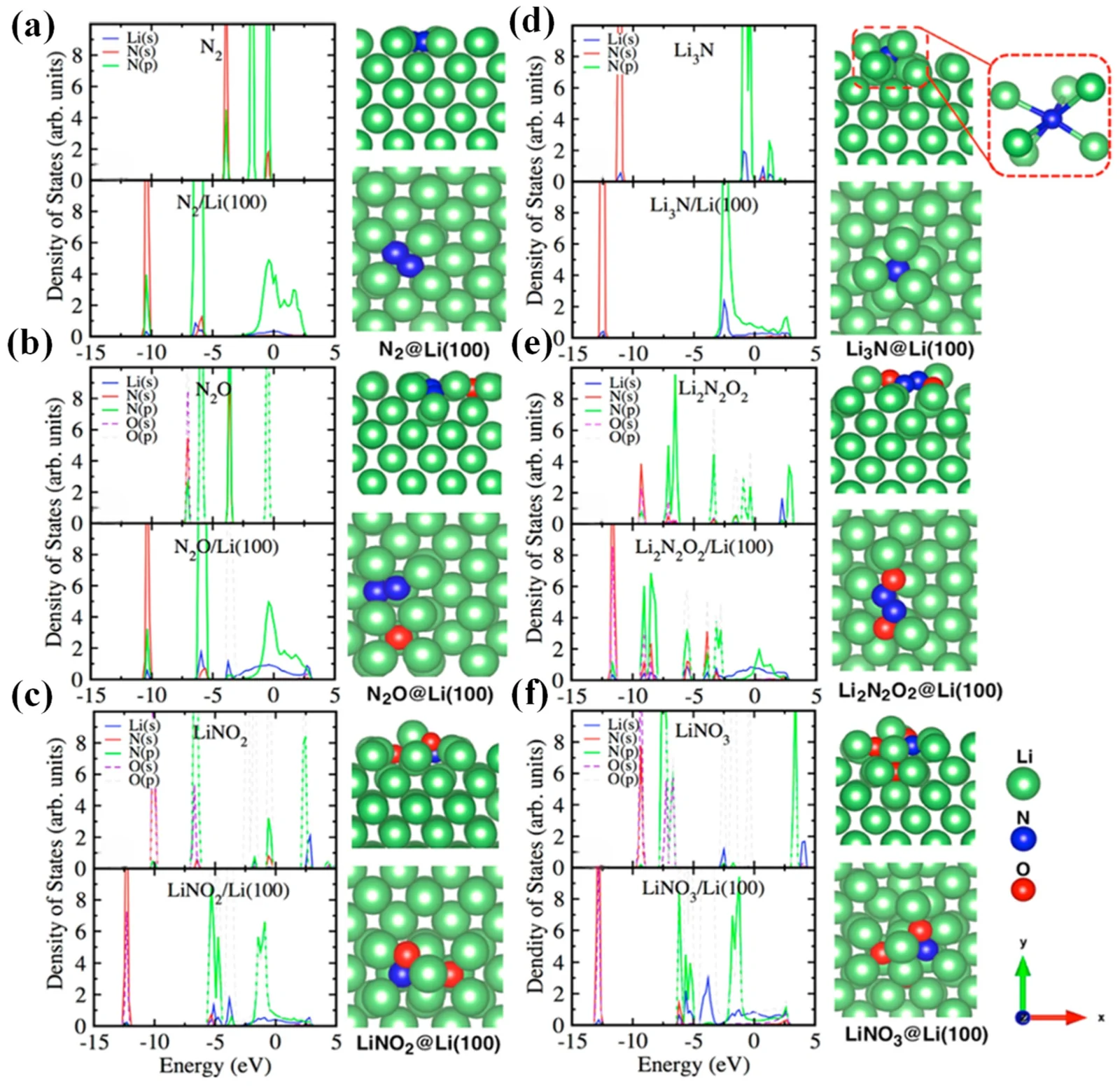
Projected densities of state (PDOS) for the gas phase and the adsorbed molecules on the Li(100) slab with the side and top views of the optimized supercells of (**a**) N_2_, (**b**) N_2_O, (**c**) LiNO_2_, (**d**) Li_3_N, (**e**) Li_2_N_2_O_2_, and (**f**) LiNO_3_ at the Li(100) slab. The Fermi level is set as the origin of the energy *x*-axis, which is shifted to zero. The DOS is scaled up five times for visualization [106]. Copyright 2017, American Chemical Society.

Figure 6 displays the PDOS of gas-phase and adsorbed molecules on the Li(100) slab, as well as the optimized supercell of the Li(100) slab. As shown in Figure 6a–e, Li_3_N molecules further form Li_6_N octahedral complexes after being adsorbed on the Li metal surface. This finding is highly consistent with other recent theoretical studies, in which the cubic Li_3_N phase composed of corner-sharing Li_6_N octahedral complexes is identified as the most stable Li_3_N phase under ambient conditions. The Li–N bond lengths before and after adsorption on the Li surface are 1.73 Å and 1.90 Å, respectively. It can be seen from Figure 6c–f that for the adsorbed Li_2_N_2_O_2_ molecules, the N–O and N–N bond lengths also increase slightly by 0.03 Å and 0.05 Å, respectively. After adsorption, LiNO_2_ and LiNO_3_ molecules dissociate into LiNO and O atoms, with the subsequent diffusion of O atoms to the Li surface. Due to the electron transfer from Li atoms to the π* orbitals of NO molecules, the N–O bond length elongates to 1.5 Å. Both electrochemical data and SEM images confirm that LiNO_3_ plays a crucial role in inhibiting the redox couple mechanism in Li–S systems. This DFT simulation was performed using the Perdew–Burke–Ernzerhof (PBE) functional and the projector augmented wave method.

**Figure 7:**
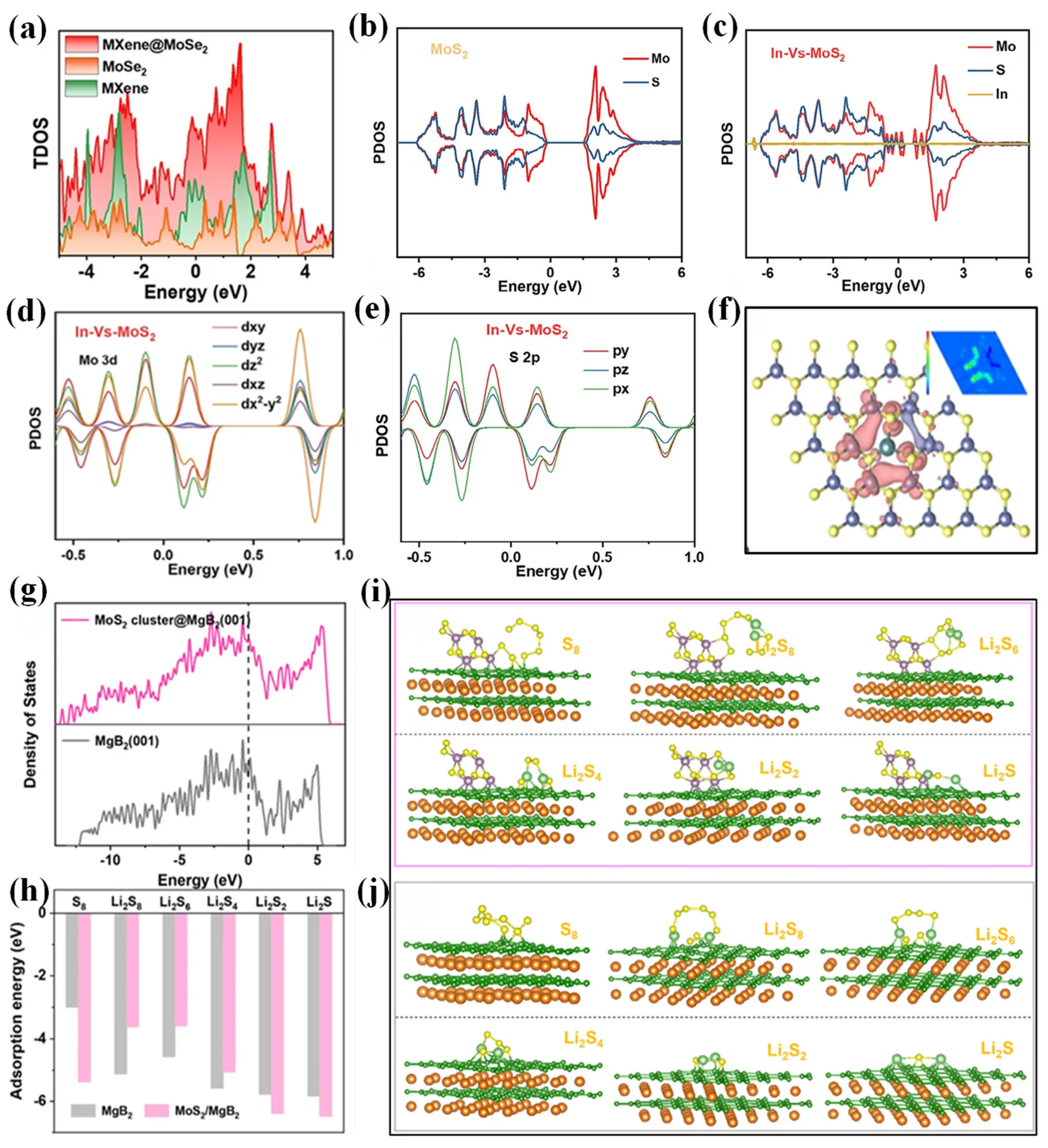
(**a**) The comparison of density of states (DOS) [97]. Copyright 2025, Wiley. (**b**,**c**) Density of states of MoS_2_ and In-Vs-MoS_2_ [110]. (**d**) PDOS of Mo-d orbital and (**e**) S-p orbital of In-Vs-MoS_2_ [110]. (**f**) The calculated 3D and 2D spin density diagrams of In-Vs-MoS_2_ [110]. Copyright 2025, Wiley. (**g**) The total density of states (DOS) of the MoS_2_ QDs@MgB_2_(001) and MgB_2_(001) [111]. (**h**) The calculated adsorption energies of S_8_ and LiPSs on MgB_2_ and MoS_2_/MgB_2_ models [111]. (**i**) The optimized adsorption structure for LiPSs on MoS_2_/MgB_2_ surface [111]. (**j**) The optimized adsorption structure for LiPSs on the MgB_2_ surface [111]. Copyright 2022, Wiley.

DFT simulations are frequently employed to calculate the band structures and density of states of chalcogenides (e.g., CoSe_2_, TiSe_2_, MoS_2_) [107], metal oxides, and nanocarbons [108]. For instance, as shown in Figure 7a, DFT calculations reveal that the MXene@MoSe_2_ heterostructure exhibits the highest DOS peak at the Fermi level, indicating a potential enhancement in charge conductivity upon heterojunction formation [109]. In addition, as illustrated in Figure 7b,c, DOS calculations demonstrate that MoS_2_ doped with S vacancies (VS) and In possesses a higher electron distribution near the Fermi level compared to pristine MoS_2_, which suggests that the vacancy and doping strategy enhances the metallicity of MoS_2_ and facilitates electron transfer. Furthermore, Figure 7d,e present a more detailed DFT analysis of the asymmetric distribution of spin-up and spin-down electron densities near the Fermi level. The spin-polarized states around the Fermi level are mainly derived from the 3dz^2^ and 3d(x^2^ − y^2^) orbitals of adjacent Mo atoms and the 3px and 3py orbitals of neighboring S atoms. Spin polarization can not only modulate the charge transfer from the catalyst surface to reaction intermediates but also provide spin electrons to accelerate the Li_2_S_2_-Li_2_S reduction catalysis at the catalytic interface [89]. The calculated 3D and 2D spin density maps of In-Vs-MoS_2_ in Figure 7f also confirm the spin polarization of surrounding Mo and S atoms induced by In doping. DFT calculations were performed in Figure 7g to further elucidate the effects of MoS_2_ quantum dots@MgB_2_ on the adsorption and catalytic conversion of lithium polysulfides. The calculated DOS of MoS_2_ QDs@MgB_2_(001) and MgB_2_(001) exhibit metallic characteristics with high electronic conductivity, which ensures high electron mobility. As shown in Figure 7h, the adsorption energy of S_8_ on MgB_2_(001) is 2.986 eV. The presence of MoS_2_ clusters promotes the interaction between S_8_ molecules and unsaturated coordinated Mo atoms, thus increasing the adsorption energy to 5.377 eV. The adsorption energies of Li_2_S_8_, Li_2_S_6_, and Li_2_S_4_ on MoS_2_/MgB_2_(001) are 3.617 eV, 3.588 eV, and 5.055 eV, respectively. In contrast, their adsorption energies on MgB_2_(001) are 5.126 eV, 4.569 eV, and 5.585 eV, respectively. The introduction of MoS_2_ on MgB_2_ is conducive to regulating the adsorption energy and adsorption configuration of LiPSs. The conversion of Li_2_S_2_ to Li_2_S plays a pivotal role in the catalytic conversion of lithium polysulfides. Evidently, the enhanced adsorption strength of LiPSs, especially Li_2_S_2_ and Li_2_S, implies that the incorporation of MoS_2_ is beneficial for suppressing the shuttle effect of LiPSs. The optimal adsorption configurations of LiPSs on MoS_2_/MgB_2_(001) and bare MgB_2_(001) are depicted in Figure 7i,j, respectively.

**Figure 8:**
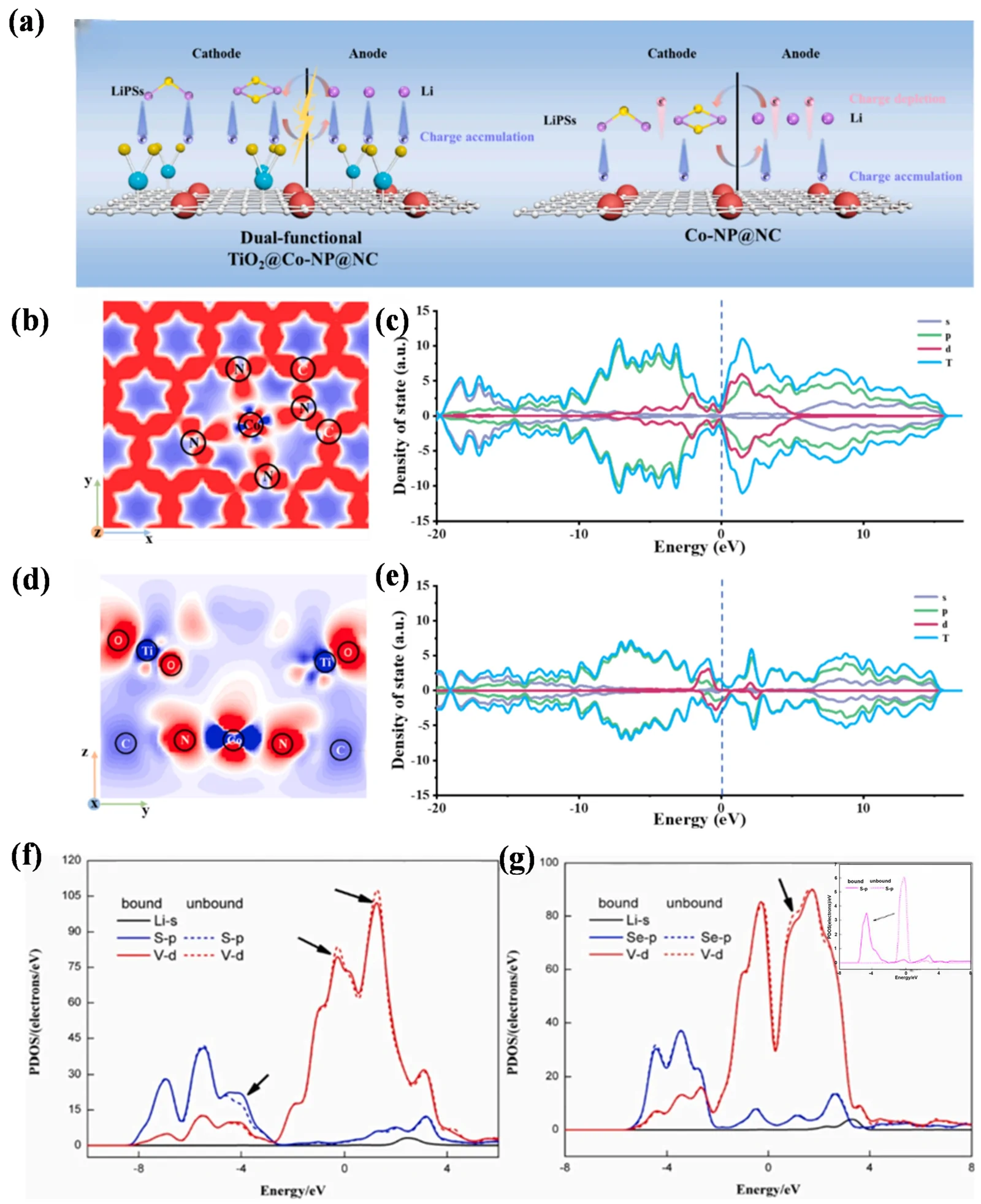
(**a**) Schematic illustration for “charge redistribution” and dual-functional electrodes in Li-S cells [114]. (**b**) x-y cross section and (**c**) y-z cross section of differential charge density redistributions in TiO_2_ @Co-NP@NC [114]. (The blue isolines represent charge accumulation and the red isolines represent charge depletion in the space). pDOS states of (**d**) TiO_2_ @Co-NP@NC and (**e**) Co-NP@NC [114]. Elsevier. The PDOS of (**f**) VS and (**g**) VSe. The embedded figure in (**g**) is the PDOS of S in Li_2_S. The dotted line and solid line represent the PDOS of VS (VSe) [115]. Elsevier.

Figure 8a–e present the fundamental effects of charge density redistribution elucidated via DFT calculations, with Figure 8a showing a schematic illustration of charge redistribution and the bifunctional electrode in lithium–sulfur batteries. Figure 8b,c demonstrate that amorphous TiO_2_ exposes additional negatively charged sites, leading to charge accumulation and depletion, and the formation of a built-in electric field on the surface of TiO_2_@Co-NP@NC.

This charge accumulation and depletion mechanism implies enhanced charge transfer between the bifunctional TiO_2_@Co-NP@NC and lithium polysulfides or Li metal. Furthermore, the stronger interactions induced by abundant negative charges can reduce the energy barriers on both the cathode and anode sides. In addition, Figure 8d,e reveal that the introduction of amorphous TiO_2_ modulates the electronic structure of the bifunctional electrode, resulting in a denser and more intense total electronic state near the Fermi level. Considering the spin-polarization effect based on the pDOS onto atomic orbitals, the variation in the total electronic band of this novel framework structure can significantly improve the electronic conductivity and catalytic performance of the full cell [100,112,113].

**Figure 9:**
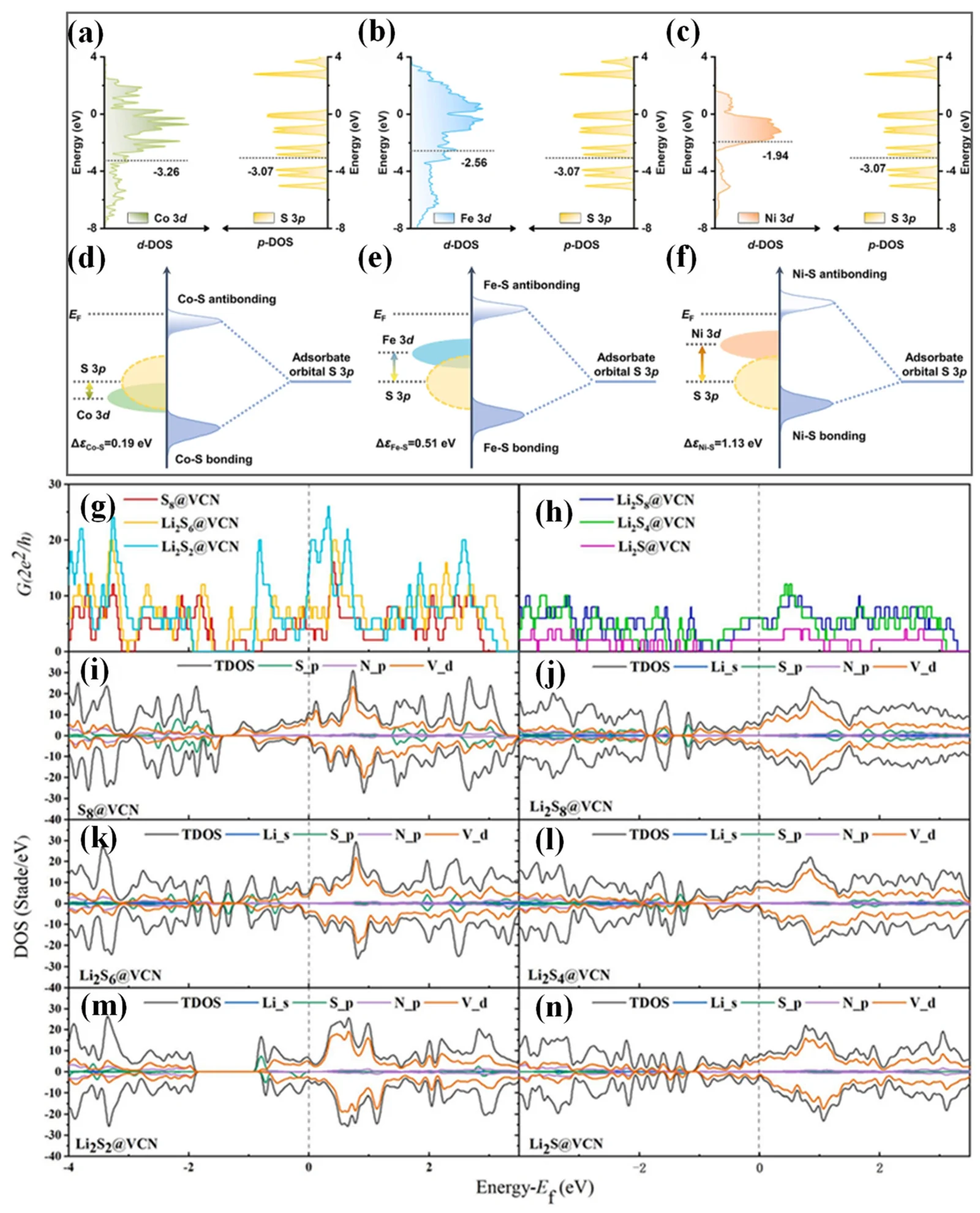
Regulation of interfacial microenvironment between TMO and LiPSs. PDOS of (**a**) Co 3d-band of Co_3_O_4_, (**b**) Fe 3d-band of Fe_2_O_3_, (**c**) Ni 3d-band of NiO, and S 3p-band of Li_2_S_4_. Schematic illustration of the coupling between S 3p of Li_2_S_4_ and (**d**) Co 3d, (**e**) Fe 3d, and (**f**) Ni 3d [116]. Copyright 2024, Elsevier B.V. (**g**,**h**) G for S_8_/Li_2_S_x_ (x = 1, 2, 4, 6, 8) adsorbed VCN. (**i**–**n**) Density of states for S_8_/Li_2_S_x_ (x = 1, 2, 4, 6, 8) adsorbed VCN [117]. Copyright 2023, Elsevier B.V.

In addition, Figure 8f,g investigate the anchoring mechanisms of S vacancies (VS) and Se vacancies (VSe) for Li_2_S, along with their corresponding pDOS. As shown in Figure 8f, the pDOS of the adsorption configuration exhibits a distinct variation after Li_2_S is anchored by VS, particularly in the V-d and S-p orbitals, which indicates significant charge transfer in the adsorption configuration and that the adsorption is dominated by VS bond interactions. For VSe, the Se-p orbitals show almost no variation; however, the obvious change in the V-d orbitals suggests charge transfer of V atoms in VSe. As illustrated in Figure 8g, the S-p orbitals undergo a variation after Li_2_S is anchored by VSe. These chalcogen vacancies (VX) exhibit excellent adsorption capacity for polysulfides, which suppresses the shuttle effect and thus contributes to the improvement of battery performance. This also highlights the applicability of DFT in guiding the rational selection of electrode materials. Figure 9a–f depict the investigation by Yuan et al. [116]. into the role of the interfacial microenvironment between transition metal oxides and lithium polysulfides in the catalytic conversion of LiPSs via DFT calculations.

Figure 9a–c show that the d-band centers of Co_3_O_4_, Fe_2_O_3_, and NiO, calculated from the individual PDOS, are −3.26 eV, −2.56 eV, and −1.94 eV, respectively, while the p-band center of the representative Li_2_S_4_ is at −3.07 eV. As illustrated in Figure 9d–f, the values of Δε(M-S) follow the trend of Δε(Co-S) (Co_3_O_4_, 0.19 eV) < Δε(Fe-S) (Fe_2_O_3_, 0.51 eV) < Δε(Ni-S) (NiO, 1.13 eV). In accordance with the Sabatier Principle, this may impede the electrocatalytic kinetics of Co_3_O_4_ and Fe_2_O_3_.

In contrast, although NiO exhibits a relatively large Δε(Ni-S), it can ensure an appropriate adsorption energy for LiPSs, which constitutes an essential prerequisite for achieving high catalytic activity in LiPSs conversion. In addition, Figure 9g,h present the G values of the VCN electrode after the adsorption of S_8_/LiPSs [117]. It can be observed that a large number of quantum channels emerge at and around the Fermi level, implying a high electrical conductivity of the adsorbed system. Furthermore, the DOS of the S_8_/LiPSs@TiCN adsorption system is calculated in Figure 9i–n. The results demonstrate that the adsorbed system still maintains excellent electrical conductivity; the high overlap between the TDOS and PDOS of VCN reveals that the electrical conductivity of the electrode is predominantly contributed by V species. Therefore, VCN can be regarded as one of the most promising cathode materials for lithium–sulfur batteries.

### 3.4. Applications for Gibbs Free Energy

The lithiation reaction thermodynamics can be investigated by calculating the Gibbs free energy of lithium–sulfide products. The reaction barrier, however, should be determined by transition state calculations rather than simply inferred from the free energy difference [118,119].

DFT-based Gibbs free energy calculations serve as a crucial theoretical foundation for elucidating the thermodynamic mechanisms and realizing rational optimization of lithium–sulfur battery systems. For different electrode or catalytic material systems, the free energy changes calculated via DFT can intuitively quantify the thermodynamic regulatory effects of materials on sulfur redox reactions [120]. The interaction between materials and sulfur species modulates the free energy landscape of the reaction, and a more negative Gibbs free energy change (ΔG) suggests a stronger thermodynamic driving force for the reaction. It should be noted that the reaction kinetics are governed by the activation free energy barrier (ΔG‡), which requires dedicated transition-state calculations to determine. Accurate calculation of Gibbs free energy using DFT constitutes a core theoretical support for advancing fundamental research and performance optimization of lithium–sulfur batteries and provides atomic-scale scientific insights for addressing the key bottlenecks in their commercialization process [121]. The shuttle effect of lithium polysulfides (LiPSs), sluggish conversion kinetics of sulfur species, and insulating nature of the solid product Li_2_S have long restricted the cycling stability and practical application of lithium-sulfur batteries [122]. However, DFT can clearly delineate the complete free energy landscape of the sulfur reduction reaction (SRR) by combining Gibbs free energy calculations with transition state searches, identify the energy states of various intermediates (from S_8_ to Li_2_S_x_, x = 8, 6, 4, 2, 1), and thus accurately locate the rate-determining step of the reaction by comparing the activation barriers of each elementary step [123]. For instance, in traditional carbon-based electrodes, the cleavage of long-chain LiPSs into short-chain species is typically accompanied by a relatively high energy barrier, while transition metal compounds or diatomic catalysts screened via DFT calculations can significantly reduce this barrier and accelerate the reaction kinetics [124].

In terms of suppressing the shuttle effect, DFT calculations provide a clear direction for material design by quantifying the adsorption free energy between electrode materials and LiPSs: physically confined materials (e.g., porous carbon, metal-organic frameworks (MOFs)) achieve weak adsorption of LiPSs through spatial confinement, whereas chemically anchored materials (e.g., MXenes, transition metal sulfides, single-atom catalysts) generate stable adsorption via the formation of strong chemical bonds (e.g., M–S, Li–N bonds), which not only prevents the diffusion and loss of LiPSs but also avoids hindering subsequent reactions due to excessively strong adsorption [125]. For example, the Fe-NC single-atom catalyst optimized via DFT calculations exhibits an adsorption free energy of −3.8 eV for Li_2_S_4_, which can efficiently capture LiPSs and simultaneously promote their further conversion [126]. Meanwhile, DFT-based Gibbs free energy calculations are also extensively applied in the optimization of catalyst activity, screening of electrolyte systems, and modification of separators [127]. In catalyst design, the reversibility of the reaction can be greatly enhanced by regulating material parameters such as the d-band center and electron transfer efficiency [128,129]. In the research of electrolytes and separators, the screening of electrolyte components that facilitate rapid Li^+^ transport and inhibit side reactions, or the design of modified separators with both ion selectivity and catalytic activity, can be realized by calculating the free energy changes in different systems, thereby further suppressing the shuttle effect [130].

Gibbs free energy can also be employed to identify the intermediates involved in the conversion process between Li_2_S_2_ and Li_2_S. Favorable intermediates exhibit an exothermic conversion from Li_2_S_2_ to Li_2_S. In the calculation of Gibbs free energy, the DFT-D3 empirical correction method is commonly adopted to characterize van der Waals (vdW) interactions. The decomposition barrier of Li_2_S, typically obtained from CI-NEB calculations as a potential energy barrier (Ea) at 0 K in vacuum, has been extensively used to evaluate the delithiation process—a lower barrier indicates more efficient kinetics.

The CI-NEB method is widely employed in DFT simulations; this approach utilizes the initial state, final state, and a series of intermediate images to identify the peak transition state on the potential energy surface, where the peak corresponds to the potential energy barrier of the entire process. Lithium–ion diffusion behavior in solid-state electrode materials has been investigated via the CI-NEB method to explore the transport kinetics, and a low diffusion barrier implies rapid ion diffusion, which contributes to the enhancement of electrochemical performance. For instance, metal sulfides can adsorb polysulfides and facilitate the subsequent conversion reactions, thus enabling efficient sulfur redox kinetics. To date, studies on the decomposition barriers of Li_2_S on nanocarbons, metals, and sulfides have demonstrated that sulfides offer greater benefits for improving battery efficiency.

**Figure 10:**
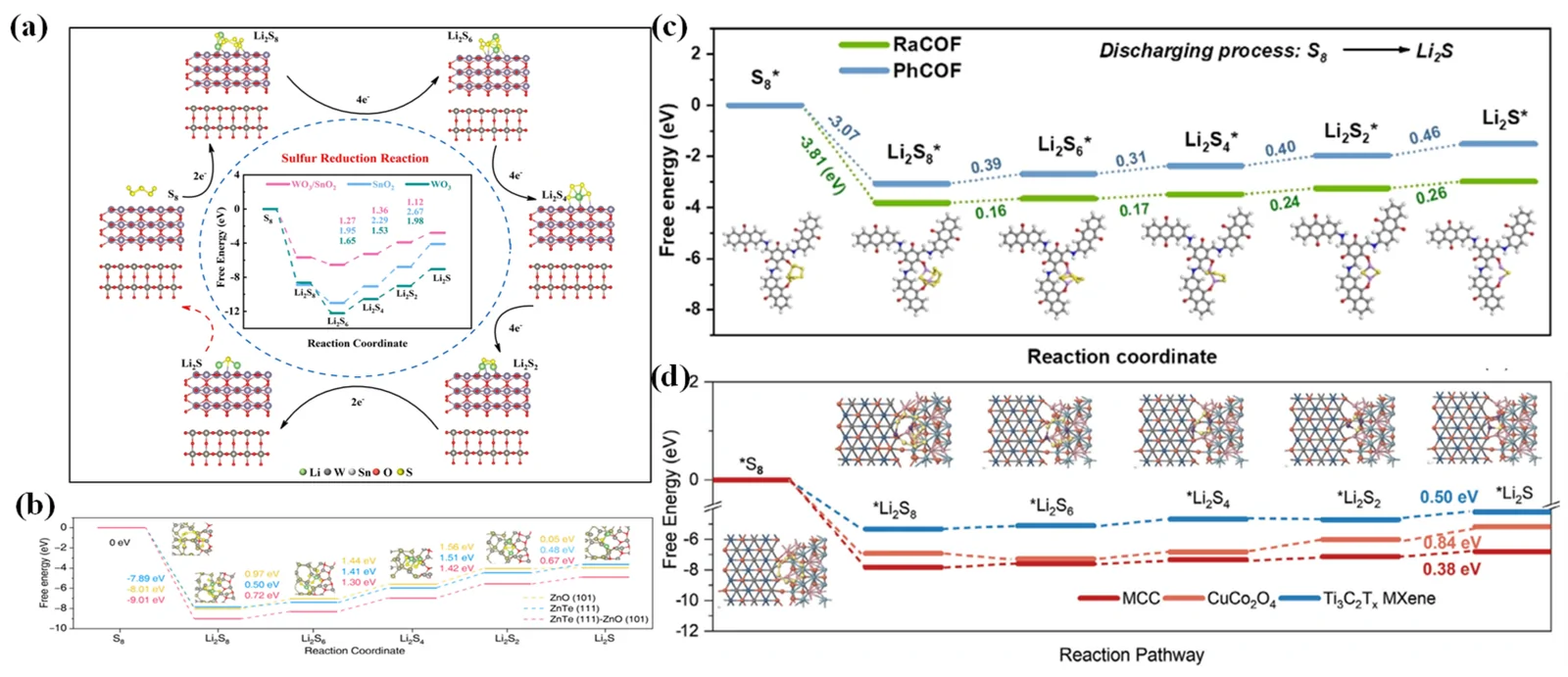
(**a**) Free energy diagrams from S to Li_2_S on different substrates (Inset shows the optimized structures of WO_3_/SnO_2_ with various sulfur species) [133]. Copyright 2025, Wiley. (**b**) Gibbs free energy of LiPSs adsorbed on the ZnO (101), ZnTe (111), and ZnO (101)-ZnTe (111) crystal facets [134]. Copyright 2025, American Chemical Society. (**c**) Stepwise free energy for the reduction process from S_8_ to Li_2_S on COF-monomer substrate (insets: the optimized configurations of RaCOF/LiPSs) [135]. Copyright 2024, Elsevier. The asterisk (*) denotes the adsorbed state on the electrode surface. (**d**) Energy profiles for the reduction of LiPSs on CuCo_2_O_4_, MXene, and MCC substrates [136]. Copyright 2024, Wiley.

DFT calculations are commonly used to investigate catalyst surfaces by calculating the Gibbs free energy changes for each reduction step from S_8_ to Li_2_S [112,131,132]. For example, Figure 10a illustrates the multi-step sulfur reduction-reaction pathway from S_8_ to Li_2_S, with the amount of electron transfer corresponding to each conversion step clearly labeled (e.g., 2e^−^ transfer for S_8_→Li_2_S_8_ and 4e^−^ transfer for Li_2_S_6_→Li_2_S_4_). The embedded curve shows the reaction free energy changes for the WO_3_/SnO_2_, SnO_2_, and WO_3_ material systems obtained from DFT calculations: by quantifying the free energy of various sulfur species (S_8_, Li_2_S_8_, Li_2_S_6_, etc.) on the surfaces of different materials; DFT plots the free energy trends along the reaction coordinate. It can be directly observed that the WO_3_/SnO_2_ composite system exhibits more gentle free energy variations, indicating that it is thermodynamically more favorable for the continuous progression of the sulfur-reduction reaction. Figure 10b presents the sulfur-reduction free energy curves for the ZnO (101), ZnTe (111), and ZnTe (111)-ZnO (101) composite systems, also derived from DFT calculations. In these calculations, the initial free energy of S_8_ was set to 0 eV to quantify the free energy of various intermediate sulfur species (Li_2_S_8_, Li_2_S_6_, etc.) and the final product Li_2_S; the free energy differences between intermediates of different systems were also compared. The results show that the ZnTe (111)-ZnO (101) composite system has a more significant decrease in free energy and lower energy barriers, demonstrating higher thermodynamic feasibility. Figure 10c presents the Gibbs free energy evolution during the sulfur reduction reaction (S_8_→Li_2_S) for two covalent organic frameworks (RaCOF and PhCOF), as calculated via DFT. This analysis directly elucidates the regulatory mechanism by which structural disparities in COFs govern the kinetics of polysulfide conversion. The comparison reveals that RaCOF exhibits a lower overall energy level than PhCOF, implying that the sulfur reduction reaction mediated by RaCOF is more thermodynamically favorable. According to the free energy calculations, the transformation from S_8_ to Li_2_S_8_ on RaCOF releases an energy of −3.81 eV, which is much more exothermic than that on PhCOF (−3.07 eV). It has been reported that the subsequent reduction steps from Li_2_S_4_ to Li_2_S_2_ and finally to Li_2_S are the rate- and capacity-determining processes in Li–S batteries, as they suffer from high activation barriers that limit the overall reaction kinetics. During the initial oxidation stage, Li_2_S undergoes delithiation preferentially. The decomposition barrier of Li_2_S, calculated as the potential energy barrier via the CI-NEB method, can be used to evaluate the difficulty of the oxidation reaction. As displayed in the below figure, the delithiation energy barriers of Li_2_S on PhCOF and RaCOF are 2.14 eV and 1.58 eV, respectively. Based on these results, RaCOF demonstrates superior electrochemical kinetic performance.

Figure 10d presents DFT calculations on the discharge process, which reveal the Gibbs free energy evolution during sulfur species transformation and further explore the catalytic process of S_8_ conversion to Li_2_S mediated by Ti_3_C_2_T_x_ MXene, CuCo_2_O_4_, and MCC (the CuCo_2_O_4_-MXene heterojunction). Based on the theoretical calculations, the energy barrier for the Li_2_S_2_→Li_2_S conversion on the CuCo_2_O_4_ surface is determined to be 0.84 eV. In contrast, this critical step is significantly reduced to 0.5 eV on the Ti_3_C_2_ Tx MXene surface, indicating that MXene offers a superior catalytic advantage for the overall transformation of lithium polysulfides (LiPSs). Notably, at the heterojunction interface constructed by CuCo_2_O_4_ and MXene, the synergistic effect of adsorption and catalytic conversion further lowers the Gibbs free energy change for the Li_2_S_2_→Li_2_S transition to 0.38 eV. This finding confirms that fully exposed MCC active sites can effectively facilitate the kinetic conversion of Li_2_S_2_ to Li_2_S.

In lithium–sulfur batteries, DFT calculations serve as a core tool for the quantification of the Gibbs free energy of all species involved in the sulfur-reduction reaction and the comparison of thermodynamic trends across different material systems, providing accurate thermodynamic insights for screening high-efficiency material systems favorable to sulfur reduction and facilitating the elucidation of the intrinsic thermodynamic mechanisms of reactions in lithium–sulfur batteries [137].

### 3.5. Application for Lithium Dendrite Inhibition and SEI Film Regulation

DFT functions as a pivotal tool for the atomic-level mechanistic exploration and rational design of lithium anodes in lithium–sulfur batteries, encompassing the targeted regulation of the solid electrolyte interphase (SEI) film and the inhibition of lithium dendrite growth. [138] It facilitates the fundamental clarification of side reactions at the sulfur species/electrolyte–lithium interfaces, remarkably reducing the cost of experimental trial-and-error and expediting advancements in battery performance [139,140]. Lithium-based liquid batteries have been investigated since the 18th century, with the corresponding technologies evolving into well-refined systems over this long timeframe [141]. These battery systems are extensively utilized in various transportation vehicles, household appliances, and other small electronic devices. Nevertheless, they are plagued by inherent drawbacks, including inadequate thermal dissipation, complex interfacial reactions, and a propensity for lithium dendrite formation at the electrolyte–electrode interface—issues that may induce short circuits or electrolyte leakage [142]. Consequently, there is an urgent requirement to develop efficient strategies for suppressing lithium dendrite growth in liquid lithium batteries [143,144]. One of the key factors contributing to capacity fading in lithium–ion batteries is the irreversible consumption of lithium species (including lithium compounds and ions), specifically the formation of irreversible lithium compounds or metallic lithium [145,146]. Irreversible metallic lithium primarily exists in the forms of dendritic lithium and dead lithium, both of which can result in the rupture of the SEI film [147,148]. The unregulated growth of lithium dendrites is therefore a critical issue accountable for the low safety performance of liquid lithium batteries [149,150]. DFT calculations provide atomic-scale mechanistic insights and precise design guidelines for addressing the core challenge of lithium dendrite inhibition in both lithium–sulfur and lithium metal batteries. By quantifying lithium deposition behavior, interfacial interactions, and the physicochemical properties of materials, DFT enables the fundamental optimization of battery systems to suppress dendrite growth. The random growth of lithium dendrites stems from the inhomogeneous deposition of lithium atoms on the electrode surface, and DFT calculations can systematically simulate the adsorption energy, diffusion pathways, and nucleation energy barriers of lithium atoms on the surfaces of different electrode materials. For instance, a comparison of the surface energies of Cu-based substrates and modified materials (e.g., MXenes, nitrogen-doped carbon, and transition metal compounds) reveals that surfaces with moderate adsorption energy (−1.5~−2.5 eV) and low diffusion energy barriers (<0.3 eV) can promote the uniform nucleation and layered growth of lithium atoms, thus avoiding the formation of dendrites from local protrusions [151]. Meanwhile, DFT can predict the formation free energy of inorganic components (e.g., LiF and Li_2_O) and the efficiency of interfacial charge transfer, which guides the design of electrolyte additives (e.g., fluorinated carbonates and LiPF_6_ derivatives) or functional separators to inhibit the inhomogeneous migration of lithium ions and electron shuttling [152].

Wang et al. [153]. identified symbiotic C-Fx surface species in the vacancies of stacked graphene (SG). Notably, the SEI film with such a functional protective structure enables stable cycling at high current densities and high lithium plating capacities in LiPF_6_/carbonate electrolytes, accompanied by enhanced Li^+^ migration and uniform, dendrite-free lithium deposition beneath the interlayer. Mo et al. [154] reported that Li^+^ is reduced to the zero-valent state at the grain boundaries and pores of solid-state electrolytes (SSEs) during diffusion, which accumulates continuously and eventually triggers short circuits. In contrast, LiBH_4_-based SSEs filled with LiF can fill the interstitial voids and exhibit low electronic conductivity, thus effectively suppressing short circuits, and the underlying mechanism has been further verified by theoretical calculations.

**Figure 11:**
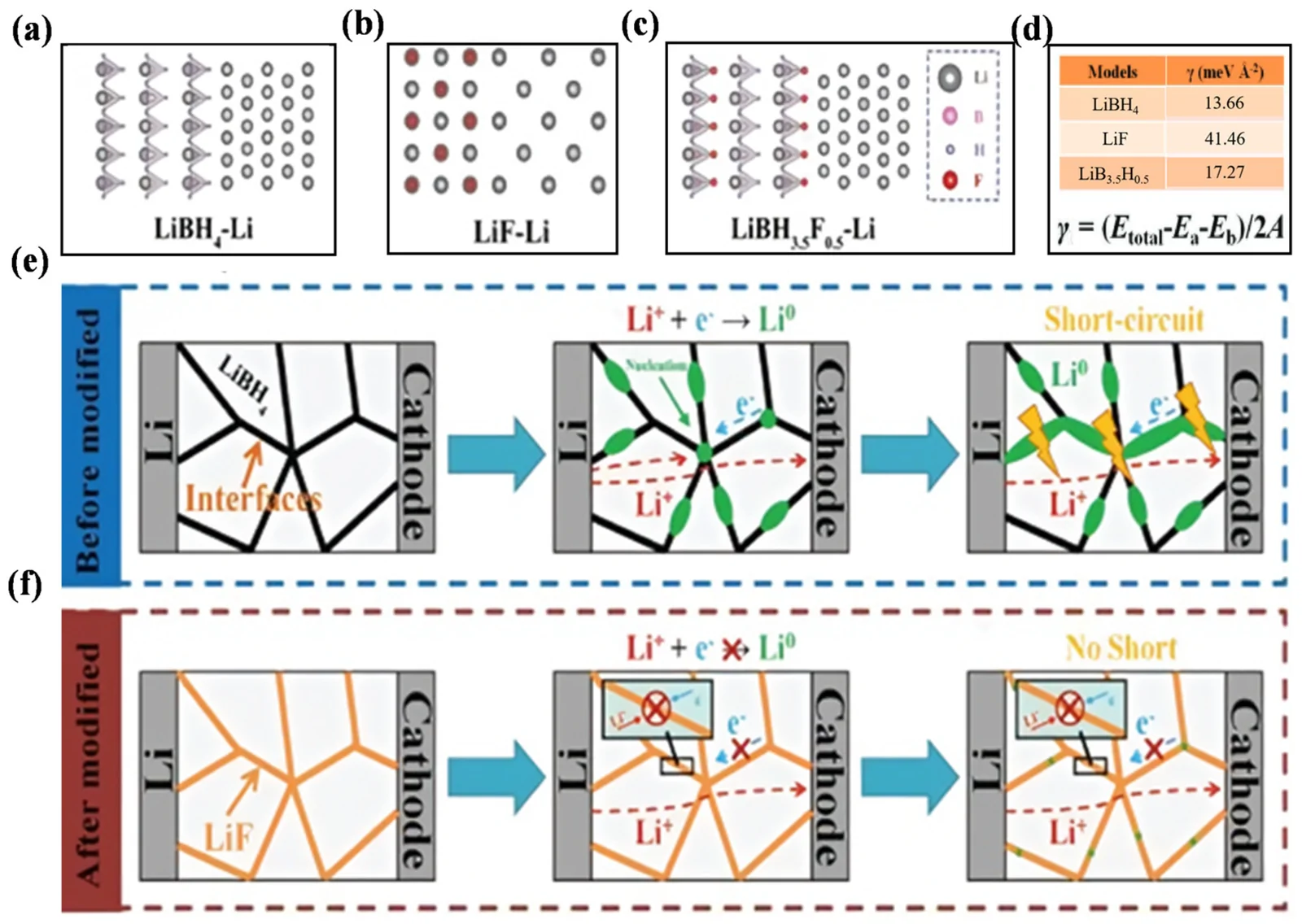
The structure of (**a**) LiBH_4_–Li, (**b**) LiF–Li, and (**c**) LiBH_3.5_F_0.5_–Li [154]. (**d**) Formation energy of different models calculated by DFT [154]. Schematic diagram of (**e**) Li dendrite formation in solid electrolyte before modified and (**f**) Li dendrite suppression mechanism in modified electrolyte system [154]. Copyright 2019, Wiley.

To gain a deeper insight into the role of LiF in inhibiting lithium dendrite formation in LiBH_4_-based electrolytes, as shown in Figure 11a–c, DFT calculations were employed to determine the formation energies between Li and different phases, including Li/LiBH_4_, Li/LiF, and Li/LiBH_4−x_F_x_. The interfacial energy of the Li/LiBH_4_ system is positive (13.66 meV), which is markedly different from the negative interfacial energies reported in oxide- and sulfide-based SSE systems, further confirming that the Li/LiBH_4_ interface is stable without chemical reactions. The formation energy of the Li/LiF system is 41.46 meV, more than three times higher than that of Li/LiBH_4_, indicating that LiF-modified LiBH_4_ can effectively inhibit lithium dendrite formation. The calculated lithium dendrite formation processes before and after LiF modification are depicted in Figure 11e,f, respectively. As shown in Figure 11e, various voids and grain boundaries exist within the pristine LiBH_4_ SSE. Li^+^ then diffuses and encounters electrons, followed by reduction to Li^0^ at the grain boundaries/voids of the SSE, which ultimately triggers short circuits. In contrast, LiF fills the voids/grain boundaries of LiBH_4_ and acts as a binder to ensure structural stability during cycling. Moreover, LiF with insulating properties can prevent the encounter between electrons and Li^+^, thus fundamentally inhibiting lithium dendrite formation.

Zhao et al. [155] demonstrated through in-depth experimental characterization and DFT calculations that LiPO_2_F_2_, a lithium salt-based electrolyte additive, can diminish the charge transfer resistance of electrodes and construct a stable fluorine-containing SEI layer on the surface of lithium metal, thereby significantly inhibiting lithium dendrite growth. Lin et al. [156] conducted DFT-based first-principles computations to ascertain the reaction energies and transfer potentials of ethylene carbonate (EC) and fluoroethylene carbonate (FEC) on the LixCoO_2_ (x = 1, 0.67) surface. Their findings indicated that FEC undergoes preferential oxidation at the initiation of the oxidative decomposition reaction, generating strongly adsorbed fluorine-containing fragments that subsequently form a dense surface layer. This dense surface layer not only facilitates the suppression of lithium dendrite growth but also enhances the cycling stability of high-voltage cathode materials utilizing FEC-based electrolytes.

**Figure 12:**
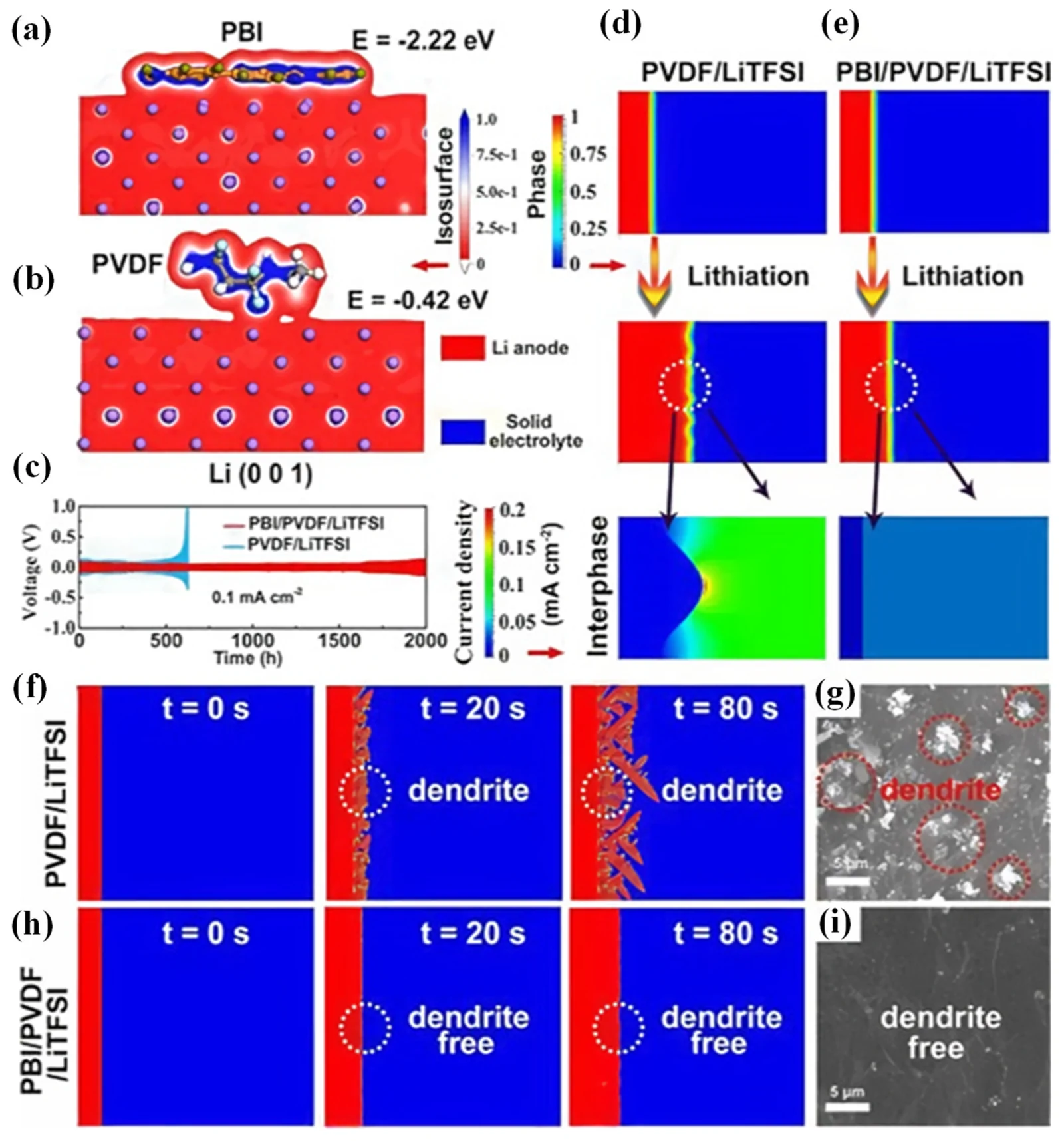
DFT calculations and phase-field simulations at interfaces. Electron density maps of (**a**) PBI and (**b**) PVDF on the Li (0 0 1) surface, respectively [157], (**c**) long-term cycling of symmetrical Li/Li cells with PBI/PVDF/LiTFSI and PVDF/LiTFSI at 0.1 mAcm^−2^ [157]. Phase-field simulations of current evolutions of (**d**) PVDF/LiTFSI and (**e**)PBI/PVDF/LiTFSI. Phase-field simulations of morphology evolutions of (**f**) PVDF/LiTFSI and (**h**) PBI/PVDF/LiTFSI.SEM images of Li metal with (**g**) PVDF/LiTFSI and (**i**) PBI/PVDF/LiTFSI after cycles [157]. Copyright 2021, Wiley.

Chen et al. [157] employed DFT and phase-field simulation methods to calculate the adsorption energy at the interfaces of PVDF/LiTFSI and polybenzimidazole (PBI)/PVDF/LiTFSI, and further simulated the current evolution process. Figure 12a,b present the electron density maps and binding energies of PBI and PVDF on the Li (001) surface obtained from DFT calculations; meanwhile, the calculated binding energy of PBI with the Li surface (E = −2.22 eV) is much lower than that of PVDF (E = −0.42 eV). A larger absolute value of the binding energy indicates stronger interfacial interaction, which confirms the more intimate interaction between PBI and the lithium surface and provides atomic–electronic scale microscopic insights for interfacial stability.

Figure 12c displays the cycling curves of symmetric Li/Li cells, where the PBI/PVDF/LiTFSI system exhibits more stable voltage and current profiles. The underlying reason for this macroscopic performance advantage is the strong interfacial interaction between PBI and the lithium surface revealed by DFT calculations. Figure 12d,e show the phase-field simulation results based on DFT-derived interfacial parameters, presenting the equipotential surfaces and interfacial phase distributions during the lithiation process of the PVDF/LiTFSI and PBI/PVDF/LiTFSI systems: the incorporation of PBI renders the interfacial equipotential surfaces more uniform and the phase distributions more regular, thus avoiding abnormal lithium deposition induced by local potential inhomogeneity. Notably, the parameter input for phase-field simulations relies on the interfacial interaction data provided by DFT calculations. The morphological evolution results from phase-field simulations in Figure 12f–h further demonstrate that lithium dendrites gradually form in the PVDF/LiTFSI system over time (t = 20 s, 80 s), whereas no dendrites are observed in the PBI-modified system even after long-term cycling. Finally, the corresponding SEM images in Figure 12g–i verify the simulation conclusions: the lithium metal surface of the PVDF/LiTFSI system after cycling exhibits distinct lithium dendrites, while no dendrites are detected on the PBI/PVDF/LiTFSI system.

The formation and degradation of the solid electrolyte interphase (SEI) have emerged as another critical issue in the design of lithium metal anodes [158,159,160,161]. Beyond the structural deformation of lithium metal, it is imperative to elucidate the mechanisms underlying SEI breakdown and formation, as the decomposition of the SEI typically results in the consumption of polysulfides [162]. To passivate and better protect the lithium metal surface against these parasitic reactions [163,164], the utilization of various additives in liquid electrolytes has become the mainstream strategy [165]. By calculating the decomposition energies and reaction pathways of electrolyte components, DFT can identify the formation products and stability of the SEI film, thus assisting in the screening of electrolytes or additives conducive to the formation of a high-quality SEI film. It can also optimize the adsorption structures between potential SEI film components (e.g., Li_3_N, LiNO_3_) and the lithium anode surface [166], as well as calculate the interfacial binding energy to evaluate the adhesion stability of the SEI film [167]. Furthermore, DFT can simulate the diffusion of lithium ions within the SEI film, balance the film’s stability and ionic conductivity, and thereby provide substantial support for the rational regulation of the SEI film.

Cao et al. [168] fabricated a 3D lithium anode with a gradient Li_3_N layer and calculated the Li^+^ diffusion energy barriers on this anode via DFT calculations. The results demonstrated that Li^+^ exhibits a low diffusion energy barrier on Li_3_N; the SEI film containing L_i3_N can facilitate the diffusive transport of Li^+^, and the Li_3_N-rich lithium anode substrate is conducive to the uniform deposition of lithium metal.

In addition, Bader charge analysis, which uses the charge within the Bader volume to represent the total charge of an atom, is a widely used DFT method for investigating the formation and cleavage of chemical bonds via charge transfer [169].

Core-level binding energy shifts obtained from DFT theoretical simulations provide an alternative approach for studying SEI film formation [170]. Upon adsorption on the lithium surface, the binding energies of LiNO_3_ and LiNO_2_ shift to lower energy levels, indicating their decomposition on the lithium anode surface. These species are commonly employed as potential components of the SEI film on lithium anodes and act as electrolyte additives to participate in SEI film formation [171]. Herein, DFT serves as a core tool for the optimization of adsorption structures and the calculation of the electronic density of states.

Previous sections have outlined how different DFT descriptors, including adsorption energy, DOS, charge density, Gibbs free energy, and reaction energy barriers, are applied independently in Li-S battery research. These parameters are not mutually exclusive; instead, they complement one another and form a hierarchical set of tools that describe interfacial interaction strength, electronic structure, charge redistribution, thermodynamic spontaneity, and kinetic reaction barriers, jointly clarifying how material modification improves the electrochemical performance of Li–S systems. Combining multiple descriptors helps avoid the drawbacks of relying on a single theoretical metric, builds robust structure–activity links between microscopic electronic features and measurable electrochemical behaviour, and yields more consistent and convincing mechanistic explanations. When studying polysulfide confinement at interfaces, adsorption energy provides a straightforward thermodynamic measure of substrate anchoring capacity and facilitates rapid screening of sulfur host materials, yet it offers little insight into the origin of interfacial interactions. Charge density analysis addresses this limitation by visualizing interfacial charge transfer, charge accumulation, and bonding characteristics, differentiating physical and chemical adsorption and explaining why adsorption energies vary across different interfaces. Together, these two descriptors enable a complete description of polysulfide anchoring that combines quantitative binding strength with mechanistic understanding, which is essential for unravelling sulfur retention and shuttle suppression. DOS/PDOS calculations extend this static interfacial picture by capturing intrinsic conductivity. Fermi level positions and orbital hybridization cannot be obtained from adsorption energy or charge density alone. However, observed orbital hybridization can cross-check bonding features identified from charge density mapping and confirm effective interfacial coupling. Additionally, DOS profiles illustrate how material engineering modulates electron transport and correlates with rate performance, offering guidance for balancing interfacial stability and electrical conductivity. Unlike these static descriptors, Gibbs free energy and reaction energy barriers target dynamic redox transformations during cycling and underpin analyses of polysulfide conversion thermodynamics and kinetics. Gibbs free energy quantifies the driving force for individual reaction steps and pinpoints rate-determining processes, while energy barriers measure kinetic hindrance and directly relate to cell polarization, reversibility and cycling durability. Such information cannot be extracted from static structural calculations. Collectively, these DFT descriptors form a logical analysis sequence: charge density and DOS expose fundamental electronic structures and interfacial bonding, adsorption energy quantifies polysulfide trapping and explains shuttle suppression, and Gibbs free energy, together with reaction barriers, describes the thermodynamics and kinetics of polysulfide conversion to interpret macroscopic trends in rate and cycling performance. Reliance on any single DFT descriptor only captures a limited aspect of the overall system and risks incomplete mechanistic interpretation. Integrating multiple descriptors into a unified framework of “electronic structure—interfacial interaction—thermodynamics—kinetics” therefore enables thorough analysis of structure–activity relationships, supporting the rational design of high-performance Li–S batteries through cathode tuning, separator modification, and electrolyte optimisation.

## 4. Summary and Perspective

Li–S batteries have emerged as a pivotal candidate for next-generation high-energy-density secondary batteries. Owing to their intrinsic advantages, including abundant sulfur resources, low cost, and environmental benignity, Li–S batteries exhibit irreplaceable application potential in new energy vehicles and large-scale energy storage systems [172,173,174]. Li–S battery technology is anticipated to expand into broader industrial applications in the coming years. However, several critical challenges still impede the practical development of Li–S batteries, such as low sulfur utilization, compromised cycling performance, severe shuttle effect of lithium polysulfides LiPSs, sulfur volume expansion upon lithiation, and lithium dendrite growth [175,176,177]. To address these bottlenecks, various strategies have been developed, including electrode modification and separator optimization. Elucidating the intrinsic working mechanisms of these strategies provides a fundamental basis for advancing the rational design of high-performance Li–S batteries [178,179]. This review focuses on the versatile applications of DFT calculations in Li–S battery research.

The GGA-PBE functional enables efficient calculation of adsorption binding energies, facilitating the investigation of sulfur host adsorption capacity for LiPSs. The DFT+U functional is employed to calculate band structures and DOS for analyzing the electronic properties of materials. The CI-NEB method can be utilized to determine the potential energy barriers of reactions, and the reaction kinetics of lithiation and delithiation have been investigated via DFT simulations. This review demonstrates that DFT methods have been widely applied to the investigation of LiPSs adsorption, as well as the electrochemical properties and reaction kinetics of cathodes in Li–S batteries. Furthermore, DFT simulations have proven effective in determining the distribution of lithium ions on lithium metal anodes. DFT analysis is frequently used to calculate charge transfer between the SEI and electrode/electrolyte interfaces, which can further reveal the formation and cleavage of chemical bonds during SEI film formation. Herein, DFT primarily serves as a core tool for optimizing adsorption structures in the above research systems.

The selection of exchange–correlation functionals is of paramount importance in DFT calculations and should be guided by the intrinsic characteristics of the system under study. For simple metallic systems with uniform electron density (e.g., pristine metallic lithium substrates), the GGA functional—such as the most commonly used PBE-GGA—is often a reasonable choice when computational efficiency is prioritized, and it can accurately predict fundamental structural properties, including bond lengths and binding energies. However, the calculation of band structures and DOS requires a precise description of electronic correlation; thus, the DFT+U functional is widely used to simulate the electronic properties of alloys, oxides, and polyanion electrodes, capturing local electronic correlations via the Hubbard-U correction term, though the choice of the U value requires careful benchmarking. For high-precision calculations of reaction energy barriers and interfacial electron transfer in Li–S batteries, meta-GGA functionals incorporating kinetic energy density variables (e.g., SCAN) offer improved accuracy for systems with strong electron localization. They provide a more elaborate description of electron localization behavior but remain computationally more demanding, and their performance depends on the specific system under study. In general, the core principle of functional selection is to accurately depict the dominant interactions within the target system. The accuracy of DFT calculations is highly dependent on the rational selection of functionals. However, it should be noted that no single functional is universally applicable, and systematic benchmarking against experimental or higher-level theoretical data is essential to ensure reliability.

In recent years, machine learning (ML) has emerged as a powerful auxiliary tool to address the low-throughput bottleneck of conventional DFT simulations in Li-S battery research. While traditional DFT calculations deliver reliable electronic and energetic information for mechanistic analysis, their high computational consumption limits large-scale screening of material candidates. By training ML models on credible DFT datasets, key parameters, including adsorption strength, electronic structure, and reaction barriers, can be efficiently predicted. The combination of DFT and ML greatly accelerates the exploration and rational design of sulfur hosts and interfacial modification materials, offering a feasible route toward high-throughput and intelligent development of advanced Li-S battery systems.

In addition to the conventional DFT descriptors and emerging data-driven strategies discussed above, several advanced DFT-based analytical methods have also gained increasing attention for deciphering intricate Li–S interfacial chemistry, despite being less summarized in existing reviews. These representative techniques include HOMO/LUMO analysis, electrostatic potential mapping, and Fukui function calculation. Specifically, HOMO/LUMO orbitals reflect the electron-donating and accepting capabilities of electrolytes and electrode materials, which helps clarify the redox activity and evolution of SEI films. Electrostatic potential mapping visually reveals surface charge distribution, enabling precise identification of preferred polysulfide adsorption sites. Meanwhile, Fukui function analysis accurately locates surface reactive sites, further interpreting the intrinsic reaction activity and selectivity of polysulfide conversion. These refined electronic descriptors can well complement conventional DFT parameters, providing multi-scale theoretical insights into SEI formation, interfacial adsorption, and redox kinetics of Li–S batteries.

In conclusion, DFT simulations demonstrate exceptional efficacy in probing key parameters—including adsorption binding energy, Gibbs free energy, and reaction energy barriers—as well as in elucidating critical interfacial processes such as lithium dendrite growth and SEI film formation. This provides robust theoretical underpinnings for fundamental investigations of Li–S batteries and offers instructive insights for the development of high-performance lithium-based battery systems. However, the majority of existing studies have centered on the cathodes and anodes of Li–S batteries, while numerous aspects of electrolyte research remain largely unexplored. For instance, recent DFT-guided studies have demonstrated the capability of computational approaches in screening electrolyte additives and elucidating interfacial decomposition mechanisms, highlighting the broader applicability of DFT beyond electrode materials [180,181].

Looking forward, several critical challenges remain for DFT-based studies of Li-S batteries. Most current calculations are performed at 0 K in vacuum, whereas practical battery operation involves finite temperatures, solvated environments, and applied electrode potentials. The development and adoption of explicit solvation models, AIMD at finite temperatures, charged interface models, and constant-potential DFT methods are urgently needed to bridge this gap. The SEI layer in Li–S batteries is largely amorphous and inhomogeneous, yet most computational models rely on crystalline or idealized structures; more realistic amorphous SEI models are required to capture the true interfacial chemistry. Functional uncertainty also remains a significant issue—different exchange–correlation functionals can yield substantially different results for the same system, and systematic benchmarking against experimental data or high-level many-body methods is essential to ensure reliability. Furthermore, DFT predictions should be closely integrated with experimental validation, such as spectroscopic characterization and electrochemical measurements, to confirm computational findings and guide practical material design. Addressing these challenges will be crucial for advancing DFT from qualitative trend analysis to quantitative predictive modeling in Li-S battery research.

## Data Availability

No new data were created or analyzed in this study. Data sharing is not applicable to this article.
